# Correlates of attendance at community engagement meetings held in advance of bio-behavioral research studies: A longitudinal, sociocentric social network study in rural Uganda

**DOI:** 10.1371/journal.pmed.1003705

**Published:** 2021-07-16

**Authors:** Bernard Kakuhikire, Emily N. Satinsky, Charles Baguma, Justin D. Rasmussen, Jessica M. Perkins, Patrick Gumisiriza, Mercy Juliet, Patience Ayebare, Rumbidzai C. Mushavi, Bridget F. O. Burns, Claire Q. Evans, Mark J. Siedner, David R. Bangsberg, Alexander C. Tsai

**Affiliations:** 1 Mbarara University of Science and Technology, Mbarara, Uganda; 2 Department of Psychology, University of Southern California, Los Angeles, California, United States of America; 3 Center for Global Health, Massachusetts General Hospital, Boston, Massachusetts, United States of America; 4 Department of Psychology, Duke University, Durham, North Carolina, United States of America; 5 Peabody College, Vanderbilt University, Nashville, Tennessee, United States of America; 6 Department of Obstetrics and Gynecology, Massachusetts General Hospital, Boston, Massachusetts, United States of America; 7 Department of Obstetrics and Gynecology, Brigham and Women’s Hospital, Boston, Massachusetts, United States of America; 8 Harvard Medical School, Boston, Massachusetts, United States of America; 9 Department of Urban Studies and Planning, Massachusetts Institute of Technology, Boston, Massachusetts, United States of America; 10 Mongan Institute, Massachusetts General Hospital, Boston, Massachusetts, United States of America; 11 Division of Infectious Diseases, Massachusetts General Hospital, Boston, Massachusetts, United States of America; 12 Oregon Health and Science University - Portland State University School of Public Health, Portland, Oregon, United States of America; University of California, San Francisco, UNITED STATES

## Abstract

**Background:**

Community engagement is central to the conduct of health-related research studies as a way to determine priorities, inform study design and implementation, increase recruitment and retention, build relationships, and ensure that research meets the goals of the community. Community sensitization meetings, a form of community engagement, are often held prior to the initiation of research studies to provide information about upcoming study activities and resolve concerns in consultation with potential participants. This study estimated demographic, health, economic, and social network correlates of attendance at community sensitization meetings held in advance of a whole-population, combined behavioral, and biomedical research study in rural Uganda.

**Methods and findings:**

Research assistants collected survey data from 1,630 adults participating in an ongoing sociocentric social network cohort study conducted in a rural region of southwestern Uganda. These community survey data, collected between 2016 and 2018, were linked to attendance logs from community sensitization meetings held in 2018 and 2019 before the subsequent community survey and community health fair. Of all participants, 264 (16%) attended a community sensitization meeting before the community survey, 464 (28%) attended a meeting before the community health fair, 558 (34%) attended a meeting before either study activity (survey or health fair), and 170 (10%) attended a meeting before both study activities (survey and health fair). Using multivariable Poisson regression models, we estimated correlates of attendance at community sensitization meetings. Attendance was more likely among study participants who were women (adjusted relative risk [ARR]_health fair_ = 1.71, 95% confidence interval [CI], 1.32 to 2.21, *p* < 0.001), older age (ARR_survey_ = 1.02 per year, 95% CI, 1.01 to 1.02, *p* < 0.001; ARR_health fair_ = 1.02 per year, 95% CI, 1.01 to 1.02, *p* < 0.001), married (ARR_survey_ = 1.74, 95% CI, 1.29 to 2.35, *p* < 0.001; ARR_health fair_ = 1.41, 95% CI, 1.13 to 1.76, *p* = 0.002), and members of more community groups (ARR_survey_ = 1.26 per group, 95% CI, 1.10 to 1.44, *p* = 0.001; ARR_health fair_ = 1.26 per group, 95% CI, 1.12 to 1.43, *p* < 0.001). Attendance was less likely among study participants who lived farther from meeting locations (ARR_survey_ = 0.54 per kilometer, 95% CI, 0.30 to 0.97, *p* = 0.041; ARR_health fair_ = 0.57 per kilometer, 95% CI, 0.38 to 0.86, *p* = 0.007). Leveraging the cohort’s sociocentric design, social network analyses suggested that information conveyed during community sensitization meetings could reach a broader group of potential study participants through attendees’ social network and household connections. Study limitations include lack of detailed data on reasons for attendance/nonattendance at community sensitization meetings; achieving a representative sample of community members was not an explicit aim of the study; and generalizability may not extend beyond this study setting.

**Conclusions:**

In this longitudinal, sociocentric social network study conducted in rural Uganda, we observed that older age, female sex, being married, membership in more community groups, and geographical proximity to meeting locations were correlated with attendance at community sensitization meetings held in advance of bio-behavioral research activities. Information conveyed during meetings could have reached a broader portion of the population through attendees’ social network and household connections. To ensure broader input and potentially increase participation in health-related research studies, the dissemination of research-related information through community sensitization meetings may need to target members of underrepresented groups.

## Abstract 2

In a social network cohort study, Emily Satinsky and colleagues estimate demographic, health, economic and social network factor associated with attendance at community sensitization meetings held in advance of participation in health-related research studies in rural Uganda.

## Author summary

**Why was this study done?:**

- Community engagement is central to the conduct of health-related research studies as a means of developing trust, increasing awareness of and engagement with research procedures, and safeguarding ethical good practice.
- Community sensitization meetings are often held in advance of global health research activities to build community awareness of key scientific and research concepts and to create opportunities for collaboration and feedback.
- Since successful research implementation requires buy-in from a range of stakeholders, fulfillment of community sensitization meeting aims requires widespread attendance and, potentially, subsequent dissemination of information to community members not in attendance.

**What did the researchers do and find?:**

- We conducted a longitudinal, sociocentric social network study with 1,630 adults in a rural region of southwestern Uganda to understand the demographic, health, economic, and social network correlates of attendance at community sensitization meetings held before 2 research study activities.
- Attendance at community sensitization meetings was more likely among study participants who were older age, women, married, members of more community groups, and living in closer geographical proximity to the meeting locations.
- Nonattendees living in the households of meeting attendees were more likely (compared with meeting attendees) to be younger age, men, unmarried, and members of fewer community groups.

**What do these findings mean?:**

- These findings suggest that information dissemination, relationships, and trust achieved through the use of community sensitization meetings held in advance of research studies may disproportionately extend to certain sociodemographic subgroups.
- However, information conveyed during community sensitization meetings may reach a broader sample of the population via informal transmission through attendees’ social network and household connections.
- Future community sensitization efforts should aim to better target members of underrepresented groups.

## Introduction

Community engagement is central to the conduct of health-related research studies. Sometimes described as stakeholder engagement [1], this process is valued by both researchers and community members (including study participants and non-study participants) for its role in cultivating trust and relationships between the research institution and the community, increasing research recruitment and retention, promoting behavior change, and safeguarding ethical good practice [2–7]. Tindana and colleagues describe community engagement as a process that aims to ensure the cultural relevance and acceptability of research procedures, minimize community disruption, avoid harm through exploitation, and consider potential ethical hazards native to the community context [8]. Through this framework, community engagement allows for a relationship that respects the community and promotes common goals and interests [2,9,10].

Research teams commonly engage with prospective study participants and their communities prior to engaging in study activities in sub-Saharan Africa [4,11–18]. Community engagement can involve diverse formats, including smaller discussions with village leaders and community advisory boards (CABs) [19–23], community mobilization, and larger meetings with community members [9,24]. Studies of these community engagement efforts have highlighted their value as a vehicle for increasing awareness and engagement of prospective study activities [25]. When employed as an intervention, community mobilization has been shown to encourage positive health behaviors, e.g., as was shown in South Africa with respect to higher uptake of HIV testing and condom use [26,27].

Large gatherings of community members, often called “community sensitization” meetings, are typically held prior to implementation of study procedures. These meetings are meant to provide information about upcoming study activities, build awareness of key scientific and research concepts [17,28,29], and create opportunities for collaboration and feedback between researchers and community members [30,31]. For example, an evaluation of a community-wide quality improvement study in rural Tanzania and Uganda elicited recommendations from village volunteers who suggested that community sensitization meetings about maternal and newborn health would support help-seeking behaviors and care practices [32]. Similarly, a qualitative study from Kenya showed that, despite a lack of awareness of mass screening and treatment for malaria after initial sensitization meetings, there was community interest in more targeted sensitization efforts [33]. By developing research literacy among potential participants [34], researchers help to ensure that the consent process is voluntary and valid [2,9,35,36] and to cultivate trust among community members [3,7,16,37,38]. A largely separate literature describes community engagement in the form of disseminating research findings back to study participants and other community members [39–44].

Power differences between research staff and participants can affect research engagement and outcomes [45,46]. By initiating sensitization meetings as guests of the community [47], researchers receive feedback from and consult with potential participants [31], thereby allowing for the co-creation of relationships that can be engaged throughout the implementation of study procedures. Through this dynamic process, sensitization meetings can help identify areas of community misinformation; establish culturally appropriate language for study materials to describe the risks, benefits, and alternatives of participation; and minimize risks to and exploitation of study participants [2,22]. Once identified, concerns about prospective study procedures can then be considered when planning for effective study implementation and/or potentially modified in response to this feedback [17,33,48–52].

Successful community sensitization and subsequent research activities require buy-in from a range of stakeholders in the community, including local political leaders, opinion leaders, and heads of households [31]. While many studies note the importance these leaders play in community sensitization efforts (e.g., to increase buy-in and attendance), and despite evidence suggesting that there is substantial ethical and instrumental value in conducting community sensitization meetings in advance of implementing research study procedures, little is known about the demographic, health, economic, and social network characteristics of community members who attend community sensitization meetings. This is an important gap in the literature because any fulfillment of meeting aims is conditioned on widespread attendance by members of the community and subsequent dissemination of the information contained therein to other community members not in attendance.

Only one study has attempted to answer this question: Dierickx and colleagues conducted a mixed-methods study in The Gambia, sampling 124 households (primary heads of households and other household leaders) representing a community of 4,456 people to assess their attendance at community sensitization meetings and elicit their perceptions about the benefits of and barriers to participating in the researchers’ study. In addition to characterizing meeting attendees, Dierickx and colleagues hypothesized that information discussed during community sensitization meetings may have been passed to nonattendees through informal means, such as word of mouth [53]. Other than this single novel study, no other study has systematically characterized nonattendees who may indirectly receive information discussed at community sensitization meetings from attendees.

To address these gaps in the literature, we aimed to estimate the environmental, demographic, health, economic, and social network correlates of attendance at community sensitization meetings. These meetings were held as part of a whole-population longitudinal sociocentric social network study in a rural region of southwestern Uganda [54]. Understanding the factors that correlate with attendance at community sensitization meetings can aid in the effective targeting of underrepresented populations for further outreach. Researchers can use this information to adapt recruitment efforts, enhance community relationships, and ultimately promote widespread awareness of and engagement with research activities, while ensuring ethical good practice.

## Methods

This study is reported following the Strengthening the Reporting of Observational Studies in Epidemiology (STROBE) guideline (S1 Checklist). The analysis was conducted using data collected between 2016 and 2019 as part of a longitudinal, sociocentric social network study in rural Uganda [54]. Study activities include surveys of every adult resident at regular intervals, along with community-wide health fairs during which clinical screening, treatment, and referral services are provided and biological specimens are obtained for research purposes. The study is conducted in a parish in Rwampara District, a rural region in southwestern Uganda. The parish is comprised of 8 villages. Most parish residents work as subsistence farmers or engage in small-scale trading/enterprise [55], and food and water insecurity are common in this rural setting [55–58].

### Community sensitization meetings

**Fig 1:**
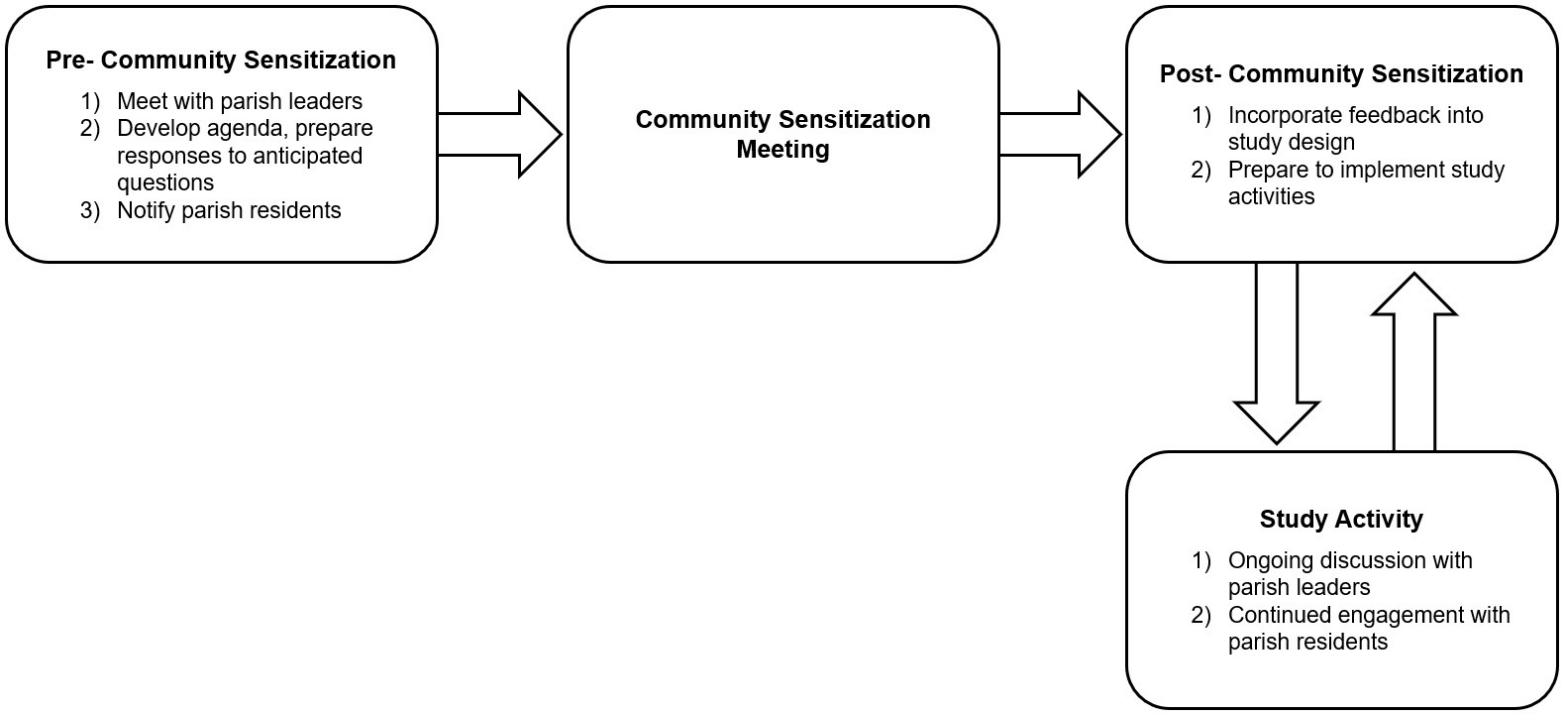
Community sensitization process.

Prior to implementation of study activities, the study team conducted a series of community sensitization meetings in each village of the parish (Fig 1). Before each meeting, the study team worked with selected community stakeholders—including local council level 1 (LC1) chairpersons (i.e., elected leaders at the lowest administrative level of Uganda’s decentralized local government system [59]), the parish chief, village health team (VHT) members, community mobilizers, key opinion leaders, religious leaders, and community development officers—to enlist their aid in mobilizing community members to attend meetings. In return for their assistance, these stakeholders were provided with 10,000 Ugandan Shillings (approximately 2.70 USD—the value of 2 kg sugar—given the exchange rate at the time the study was conducted). Community mobilization efforts include distributing placards and banners, broadcasting announcements on the radio, and making written/verbal announcements during community meetings and religious gatherings in local churches and mosques.

In preparation for these meetings, our study team first convened to develop an agenda, brainstorm questions that would likely be raised by community members, and prepare responses. One or 2 community sensitization meetings were held in each village, approximately 1 to 2 months before commencement of subsequent study procedures. Meetings were conducted in Runyankore, the local language, and generally lasted between 1 and 2 hours.

While there were slight variations from site to site, in general, each meeting followed a similar agenda. Attendees were asked to sign a logbook upon arrival. Once attendees were seated, the meetings began with an opening prayer and, depending on the size of the meeting, introductions. Attendees were provided with soda and cake to enjoy during the meetings. Following introductions, the study team explained the upcoming study procedures and provided general information about its purposes and potential risks, benefits, and alternatives. The information shared at community sensitization meetings was intended to provide a general overview of study procedures, with the expectation that more detailed information would be provided, on a one-on-one basis, during the informed consent process. Following the presentation of the upcoming study activities, attendees were given opportunities to ask questions, share concerns, and provide guidance.

### Ethical approval and integration of community feedback

Prior to commencing the study, we obtained feedback from a CAB comprised of 8 community leaders, including 4 women and the district development officer. Their feedback was incorporated into the study design, and the study protocol was reviewed and approved by the Mbarara University of Science and Technology Research Ethics Committee and the Partners Human Research Committee. Consistent with Ugandan national guidelines, clearance for the study was also obtained from the Uganda National Council for Science and Technology. Upon receiving approval, we began conducting community sensitization meetings. Additional community sensitization meetings were held prior to subsequent waves of the community surveys and the community health fairs, thus providing opportunities for community input to be incorporated into subsequent research study activities. For example, during a community sensitization meeting held prior to the first community health fair, meeting attendees requested that our study team provide cervical cancer screening as part of the community health fair activities. In response, we incorporated into the subsequent community health fair a program of cervical cancer prevention education, high-risk human papillomavirus testing, and screening for premalignant lesions using visual inspection with acetic acid (with cryotherapy for screen-positive women meeting treatment criteria). For all study activities, participants provided written informed consent prior to participating; those who could not read and/or write were permitted to indicate consent with a thumbprint mark.

### Primary outcome variable

**Fig 2:**
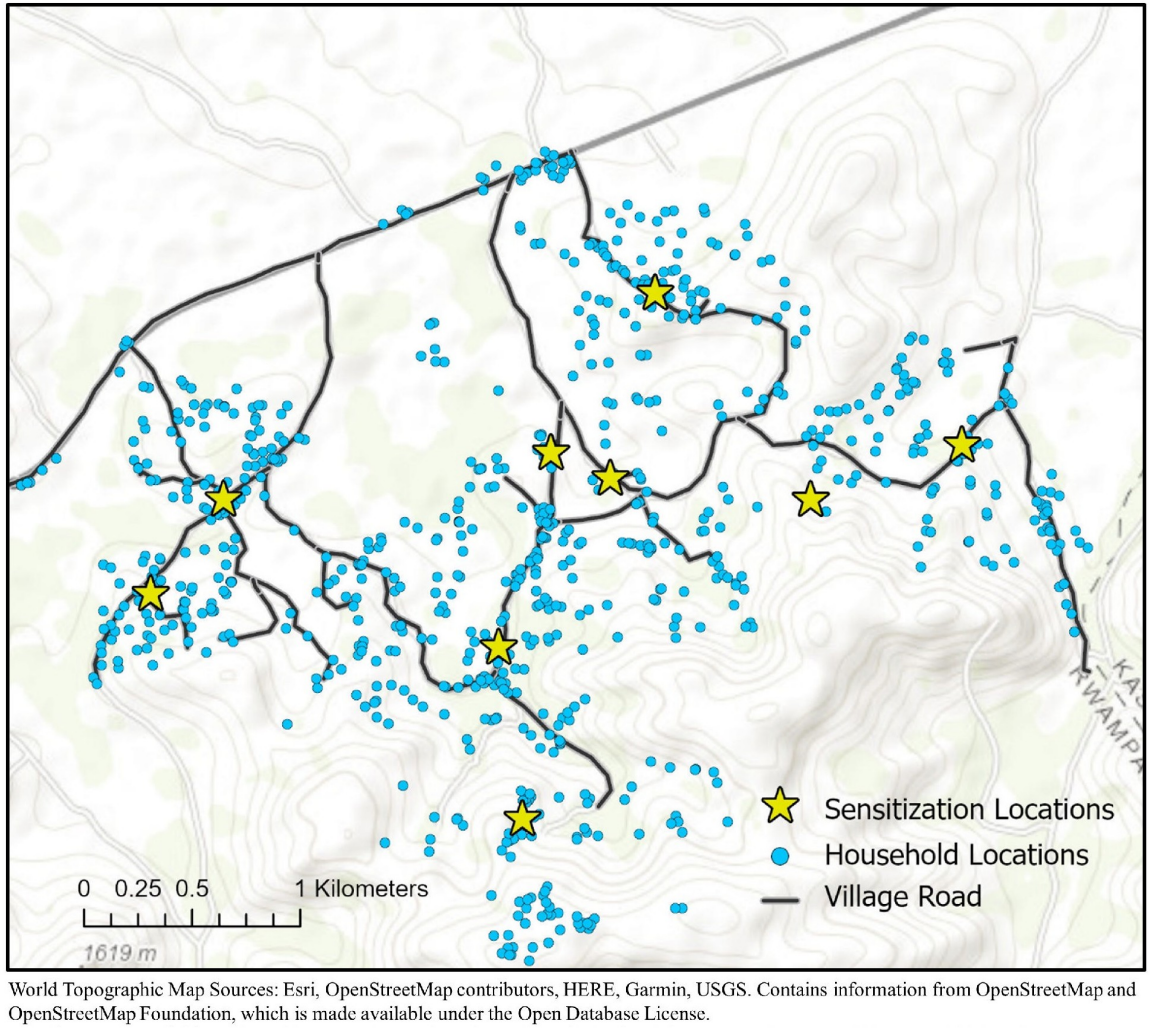
Map of the study parish, community sensitization meeting locations, and participants’ households. Base layer map available at: www.arcgis.com/apps/mapviewer/index.html?layers=30e5fe3149c34df1ba922ebbf808f.

The primary outcome for the present study was attendance at community sensitization meetings. This information was recorded from attendance logs collected from 8 community sensitization meetings held before the community survey and from 16 community sensitization meetings held before the community health fair (Table 1, Fig 2). From these data, we created 2 dichotomous outcome variables specifying attendance or nonattendance at the community sensitization meetings: (1) attendance at any community survey sensitization meeting; and (2) attendance at any community health fair sensitization meeting. We were unable to find the attendance log for one of the community sensitization meetings (held in Bukuna 1 prior to the community survey).

**Table 1:** Sensitization meetings before each study activity.

Villages	Community Survey	Community Health Fair	Total
Buhingo	1	2	**3**
Bukuna 1	*	2	**2**
Bukuna 2	1	2	**3**
Bushenyi	1	2	**3**
Nyakabare	1	2	**3**
Nyamikanja 1	1	2	**3**
Nyamikanja 2	1	1	**2**
Rwembogo	1	2	**3**
Parish Headquarters		1	**1**
Parish Leadership	1		**1**
**Total**	**8**	**16**	**24**

*One meeting was held in this village but was excluded from analysis due to missing attendance log.

### Explanatory variables

All attendee names were matched with participants’ unique study identification numbers to facilitate linkage of the 2018 to 2019 attendance variables to study participant data collected during the previously administered (2016 to 2018) community survey. Variables used in this study represented environmental, demographic, health, economic, and social network domains. Using household Global Positioning System coordinates, we calculated the shortest straight-line distance (in kilometers) from each study participant’s home to the meeting location in their respective village (continuous). Demographic variables included sex (binary), age (continuous), marital status (binary), and educational attainment (binary). Health variables included self-reported HIV serostatus (binary), obesity (binary; based on waist circumference, measured halfway between the lower costal margin and the iliac crest, with thresholds of ≥102cm for men and ≥88 cm for women who were not currently pregnant [60]), and depression symptom severity (continuous; modified Hopkins Symptom Checklist for Depression [58,61,62]). Economic variables included food insecurity (categorical; 9-item Household Food Insecurity Access Scale [55,63]), water insecurity (categorical; 8-item Household Water Insecurity Access Scale [57,58]), and household asset wealth (categorical; [64,65]).

Survey data were used to capture different components of study participants’ social integration, or their participation in various aspects of community life [66,67]. We administered network name generators [68] to elicit social ties: Each participant was asked to name specific residents in the parish (“alters”) with whom they interacted on a regular basis. We used 5 different domain-specific name generators (social, health, financial, emotional, and food exchange) to ensure that participants identified alters across multiple domains of personal life [69–71]. These data were used to calculate individual network characteristics, including in-degree, out-degree, closeness centrality, and betweenness centrality [72]. We used a locally derived 10-item scale to measure membership and participation in different community groups (continuous). Finally, the 3-item University of California at Los Angeles Loneliness Scale [73] was used to assess study participants’ subjective experiences of connectedness (continuous).

### Data analysis

The analysis was not preregistered, but we followed a prespecified analysis plan and tracked deviations that resulted from peer review (S1 Text). Single-variable and multivariable Poisson regression models with robust estimates of variance were fitted to estimate correlates of attendance at community sensitization meetings held before the community survey and the community health fair (2018 to 2019). Explanatory variables were based on data collected during the previously administered community survey (2016 to 2018). We adjusted for clustering at the village level. We fitted separate regression models for the 2 dichotomous attendance variables since we hypothesized that participants from different sociodemographic subgroups might be interested in attending meetings before the study activities for different reasons. For example, the community health fair provided a clear service (i.e., disease screening/testing and referral for treatment), so individuals with health concerns or individuals whose family members had health problems might be more likely to attend. As described previously, we were unable to find the attendance log for the community sensitization meeting held in Bukuna 1 prior to the community survey. Therefore, models estimating correlates of attendance at a meeting before the community survey excluded residents from that village. Following Zou, the exponentiated regression coefficients were interpreted as relative risk ratios [74].

The sociocentric social network design of the cohort permitted us to identify study participants who did not attend a community sensitization meeting themselves but who may have been indirectly exposed to a meeting (and the information disseminated therein) through their social affiliation with someone who did attend a meeting. We identified the number of study participants who did not attend a community sensitization meeting themselves and who were a geodesic distance of 1 from meeting attendees [75]. Formally, the geodesic distance between 2 vertices in a network graph is the number of edges corresponding to the shortest path connecting the 2 vertices; informally, a geodesic distance of 1 is one handshake away. These participants did not attend a community sensitization meeting themselves but were nominated as an alter (in the previously administered community survey), across any of the 5 domains, by someone who had attended a meeting. Similarly, we used household roster data to identify the number of study participants who did not attend a community sensitization meeting themselves and who lived in the same household with at least 1 meeting attendee. Thus, we were able to identify the number of study participants who were directly exposed to a meeting (i.e., attendees) and the number of study participants who may have been indirectly exposed to a meeting (i.e., nonattendees who were either one handshake away from an attendee or who resided in the same household with an attendee). The total sum of directly exposed and indirectly exposed study participants provided us with an estimate of the potential reach of the information conveyed during the community sensitization meetings.

Analyses were conducted using Stata version 16 (College Station, Texas).

## Results

### Characteristics of the sample

Of 1,795 individuals eligible for the community survey, 1,630 individuals participated (response rate, 91%; Table 2). Of these participants, 264 (16%) attended a community sensitization meeting before the community survey, 464 (28%) attended a meeting before the community health fair, 558 (34%) attended a meeting before either study activity (survey or health fair), and 170 (10%) attended a meeting before both study activities (survey and health fair). Of note, 56 additional individuals attended a community sensitization meeting but did not participate in the prior community survey (either because they refused, were not found, or were not yet enrolled in the study). These meeting attendees were therefore excluded from the analyses. Yet overall, 281 (16%) parish residents attended a meeting before the community survey, 510 (28%) parish residents attended a meeting before the community health fair, 614 (34%) parish residents attended a meeting before either study activity, and 177 (10%) parish residents attended at least 1 meeting before both study activities. Attendance at the meetings held before the community survey averaged 36 attendees (range, 10 to 61), while the meetings held before the community health fair averaged 43 attendees (range, 24 to 70).

**Table 2:** Characteristics of study participants stratified by attendance or nonattendance at any community sensitization meeting.

	Attendance at Any Community Sensitization Meeting					
	Attended Meeting (*n =* 558, 34.2%)		Did Not Attend Meeting (*n* = 1,072, 65.8%)		Total (*N =* 1,630)	
	n	%	n	%	n	%
**Age Category**						
18–25 years	56	10.0%	309	28.8%	365	22.4%
26–35 years	129	23.1%	276	25.8%	405	24.9%
36–45 years	116	20.8%	185	17.3%	301	18.5%
46–55 years	132	23.7%	132	12.3%	264	16.2%
56+ years	117	21.0%	154	14.4%	271	16.6%
Missing	8	1.43%	16	1.49%	24	1.47%
**Sex**						
Female	379	67.9%	532	49.6%	911	55.9%
Male	179	32.1%	540	50.4%	719	44.1%
**Married**						
Yes	392	70.3%	602	56.2%	994	61.0%
No	166	29.8%	470	43.8%	636	39.0%
**Education**						
Completed Primary School	281	50.4%	697	65.0%	978	60.0%
Did Not Complete Primary School	277	49.6%	375	35.0%	652	40.0%
**HIV Status**						
HIV Positive	68	12.1%	99	9.24%	167	10.3%
HIV Negative	490	87.8%	973	90.8%	1,463	89.8%
**Obese**						
Yes	205	36.7%	334	31.2%	539	33.1%
No	326	58.4%	702	65.5%	1,028	63.1%
Missing	27	4.84%	36	3.36%	63	3.87%
**Depression**						
Median (IQR)	1.40 (1.20–1.73)		1.33 (1.20–1.67)		1.40 (1.20–1.67)	
**Household Food Insecurity**						
Food secure	159	28.5%	366	34.1%	525	32.2%
Mild food insecurity	59	10.6%	148	13.8%	207	12.7%
Moderate food insecurity	238	42.7%	382	35.6%	620	38.0%
Severe food insecurity	101	18.1%	165	15.4%	266	16.3%
Missing	1	0.18%	11	1.03%	12	0.74%
**Household Water Insecurity**						
Water secure	258	46.2%	536	50.0%	794	48.7%
Mild water insecurity	72	12.9%	118	11.0%	190	11.7%
Moderate food insecurity	129	23.1%	222	20.7%	351	21.5%
Severe food insecurity	98	17.6%	184	17.2%	282	17.3%
Missing	1	0.18%	12	1.12%	13	0.80%
**Membership in Community Groups (No. of Groups)**						
Median (range)	1 (0–9)		0 (0–6)		1 (0–9)	
**Participation in Community Groups (No. of Groups)**						
Median (range)	1 (0–8)		0 (0–6)		0 (0–8)	
**Loneliness**						
Median (IQR)	3 (3–5)		3 (3–5)		3 (3–5)	
**Distance to Meetings in Village (km)**						
Median (IQR)	0.36 (0.23–0.50)		0.40 (0.27–0.63)		0.39 (0.25–0.59)	
**In-Degree**						
Median (IQR)	6 (3–9)		3 (1–6)		4 (2–7)	
**Out-Degree**						
Median (IQR)	6 (4–8)		5 (3–7)		5 (4–7)	
**Closeness Centrality**						
Median (IQR)	0.24 (0.22–0.25)		0.23 (0.21–0.24)		0.23 (0.22–0.25)	
**Betweenness Centrality**						
Median (IQR)	5,472 (2,099–12,585)		2,808 (444–7,605)		3,708 (862–9,318)	

Figures do not add to 100% due to rounding.

IQR, interquartile range.

Compared to the full sample of participants (mean age = 39.4 years; standard deviation [SD] = 16.7), participants who attended community sensitization meetings were older (community survey: mean age = 45.1, SD = 15.6; community health fair: mean age = 44.7, SD = 15.5). Additionally, a higher percentage of meeting attendees were women (overall sample, 56%; community survey meeting attendees, 65%; community health fair meeting attendees, 70%) and married (overall sample, 61%; community survey meeting attendees, 75%; community health fair meeting attendees, 70%).

### Correlates of attendance at community sensitization meetings

In the single-variable Poisson regression models, adjusted for clustering at the village level, the following variables had statistically significant associations with attendance at a community sensitization meeting: older age, female sex, being married, not having completed primary school, higher depression symptom severity, membership and participation in more community groups, and residing in closer geographic proximity to the meeting location (Table 3). Attendance at community sensitization meetings was more likely among study participants who were older age (adjusted relative risk [ARR]_survey_ = 1.02 per year, 95% confidence interval [CI], 1.01 to 1.02, *p* < 0.001; ARR_health fair_ = 1.02 per year, 95% CI, 1.01 to 1.02, *p* < 0.001), married (ARR_survey_ = 1.74, 95% CI, 1.29 to 2.35, *p* < 0.001; ARR_health fair_ = 1.41, 95% CI, 1.13 to 1.76, *p* = 0.002), and members of more community groups (ARR_survey_ = 1.26 per group, 95% CI, 1.10 to 1.44, *p* = 0.001; ARR_health fair_ = 1.26 per group, 95% CI, 1.12 to 1.43, *p* < 0.001). Attendance at community sensitization meetings was less likely among study participants who lived farther away from meeting locations (ARR_survey_ = 0.54 per kilometer, 95% CI, 0.30 to 0.97, *p* = 0.041; ARR_health fair_ = 0.57 per kilometer, 95% CI, 0.38 to 0.86, *p* = 0.007).

**Table 3:** Unadjusted and multivariable Poisson regression models estimating correlates of attendance at community sensitization meetings.

	Attendance at Community Sensitization Meeting Held in Advance of Community Survey				Attendance at Community Sensitization Meeting Held in Advance of Community Health Fair			
	RR (95% CI)	*p*-value	ARR (95% CI)	*p*-value	RR (95% CI)	*p*-value	ARR (95% CI)	*p*-value
**Age**	1.02 (1.01–1.02)	<0.001	1.02 (1.01–1.02)	<0.001	1.02 (1.01–1.02)	<0.001	1.02 (1.01–1.02)	<0.001
**Female**	1.45 (1.10–1.91)	0.009	1.38 (0.99–1.92)	0.055	1.85 (1.41–2.41)	<0.001	1.71 (1.32–2.21)	<0.001
**Married**	1.79 (1.31–2.46)	<0.001	1.74 (1.29–2.35)	<0.001	1.48 (1.20–1.83)	<0.001	1.41 (1.13–1.76)	0.002
**Completed Primary School**	0.68 (0.53–0.88)	0.003	0.94 (0.78–1.14)	0.551	0.71 (0.58–0.86)	<0.001	1.05 (0.86–1.28)	0.627
**HIV Positive**	1.13 (0.66–1.94)	0.662	1.11 (0.59–2.11)	0.746	1.25 (0.93–1.69)	0.144	1.14 (0.89–1.47)	0.302
**Obese**	1.20 (0.92–1.56)	0.175	0.94 (0.73–1.21)	0.615	1.35 (1.11–1.65)	0.003	0.99 (0.87–1.14)	0.911
**Depression Symptom Severity**	1.37 (1.14–1.65)	0.001	1.29 (0.98–1.69)	0.072	1.25 (1.10–1.43)	0.001	1.08 (0.90–1.30)	0.424
**Household Food Insecurity**: Food Secure (reference)								
Mild Food Insecurity	1.16 (0.79–1.70)	0.451	0.88 (0.52–1.50)	0.633	0.98 (0.70–1.35)	0.881	0.87 (0.63–1.20)	0.408
Moderate Food Insecurity	1.28 (0.73–2.25)	0.381	0.98 (0.51–1.88)	0.952	1.30 (0.99–1.70)	0.060	1.06 (0.80–1.40)	0.693
Severe Food Insecurity	1.22 (0.76–1.95)	0.402	0.93 (0.55–1.56)	0.771	1.28 (1.02–1.59)	0.030	1.07 (0.84–1.35)	0.587
**Household Water Insecurity**: Water Secure (reference)								
Mild Water Insecurity	1.30 (0.99–1.70)	0.056	1.09 (0.92–1.29)	0.327	1.06 (0.89–1.26)	0.485	0.93 (0.81–1.08)	0.343
Moderate Water Insecurity	1.07 (0.70–1.64)	0.754	0.94 (0.63–1.39)	0.745	1.11 (0.95–1.30)	0.199	0.99 (0.87–1.14)	0.909
Severe Water Insecurity	0.91 (0.63–1.31)	0.594	0.81 (0.63–1.05)	0.115	1.01 (0.81–1.26)	0.923	0.90 (0.75–1.08)	0.244
**Household Asset Wealth Quintile Category** 1st (Poorest, reference)								
2nd	1.17 (0.79–1.71)	0.434	1.08 (0.69–1.71)	0.731	1.01 (0.86–1.19)	0.906	0.95 (0.81–1.11)	0.541
3rd	1.09 (0.91–1.30)	0.357	1.01 (0.80–1.26)	0.962	1.04 (0.96–1.12)	0.320	0.97 (0.83–1.14)	0.747
4th	1.00 (0.85–1.16)	0.957	0.86 (0.63–1.18)	0.348	0.92 (0.76–1.11)	0.393	0.81 (0.67–0.98)	0.033
5th (Least poor)	0.62 (0.37–1.05)	0.079	0.61 (0.34–1.10)	0.100	0.58 (0.41–0.81)	0.001	0.57 (0.41–0.80)	0.001
**Membership in Community Groups (No.)**	1.34 (1.26–1.43)	<0.001	1.26 (1.10–1.44)	0.001	1.33 (1.23–1.43)	<0.001	1.26 (1.12–1.43)	<0.001
**Participation in Community Groups (No.)**	1.34 (1.21–1.47)	<0.001	1.03 (0.82–1.30)	0.809	1.31 (1.22–1.40)	<0.001	0.99 (0.93–1.06)	0.840
**Loneliness**	1.01 (0.93–1.10)	0.796	0.99 (0.91–1.07)	0.798	1.01 (0.98–1.05)	0.377	0.99 (0.96–1.02)	0.657
**Distance to Meetings in Village (km)**	0.51 (0.28–0.93)	0.028	0.54 (0.30–0.97)	0.041	0.56 (0.39–0.80)	0.002	0.57 (0.38–0.86)	0.007
**Constant**			0.04 (0.02–0.09)	<0.001			0.08 (0.05–0.15)	<0.001
**Observations**			1,297				1,524	

ARR, adjusted relative risk; CI, confidence interval; RR, relative risk.

The adjusted models include each of the variables listed in the table row headers: age, sex, marital status, education, HIV status, obesity, depression symptom severity, household food insecurity, household water insecurity, household asset wealth, membership in community groups, participation in community groups, loneliness, and straight-line distance to the meeting corresponding to participant’s village of residence.

There were slight differences in some of the estimated associations in terms of convention thresholds of statistical significance. For example, women were more likely to have attended a community sensitization meeting before the health fair (ARR = 1.71, 95% CI, 1.32 to 2.21, *p* < 0.001) but not more likely to have attended a community sensitization meeting held before the community survey (ARR = 1.38, 95% CI, 0.99 to 1.92, *p* = 0.055). Furthermore, compared with individuals from the poorest household asset wealth quintile category, the least poor individuals were less likely to have attended a community sensitization meeting before the health fair (ARR = 0.57, 95% CI, 0.41 to 0.80, *p* = 0.001); the least poor individuals were similarly less likely to have attended a community sensitization meeting before the community survey, but the estimated association was not statistically significant (ARR = 0.61, 95% CI, 0.34 to 1.10, *p* = 0.10).

### Expanded reach of community sensitization meetings beyond attendees

In addition to the 264 study participants who attended a sensitization meeting before the community survey, an additional 533 (33%) participants had a geodesic distance of 1 from at least one of the attendees (Table 4). The remaining 833 (51%) study participants were more than 1 geodesic from an attendee. Compared with the nonattendees who were not part of attendees’ social networks, nonattendees who were part of attendees’ social networks were more likely to be older age (44.7 years versus 35.3 years, difference, 9.4 years, 95% CI, 7.7 to 11.2, *p* < 0.001). Additionally, nonattendees in attendees’ social networks were more likely to be women (57.8% versus 51.9%, χ^2^ = 4.60, *p* = 0.032), married (70.9% versus 50.2%, χ^2^ = 57.5, *p* < 0.001), and members of more community groups (1.13 groups versus 0.63 groups, difference, 0.50 groups, 95% CI, 0.39 to 0.60, *p* < 0.001) (S1 Table).

**Table 4:** Reach of community sensitization meetings through attendees’ social networks and households.

	Attendance at Any Community Sensitization Meeting Before			
	Community Survey		Community Health Fair	
	n	%	n	%
	**Social Network Reach**			
Attendees	264	16.2%	464	28.5%
Nonattendees in Attendees’ Social Network	533	32.7%	593	36.4%
**Total Possible Social Network Reach**	**797**	**48.9%**	**1,057**	**64.8%**
Nonattendees not in Attendees’ Social Network	833	51.1%	573	35.2%
	**Household Reach**			
Attendees	264	16.2%	464	28.5%
Nonattendees in Attendees’ Households	281	17.2%	433	26.6%
**Total Possible Household Reach**	**545**	**33.4%**	**897**	**55.0%**
Nonattendees not in Attendees’ Households	1,085	66.6%	733	45.0%
	**Combined Social Network and Household Reach**			
Attendees	264	16.2%	464	28.5%
Nonattendees in Attendees’ Social Networks only	368	22.6%	339	20.8%
Nonattendees in Attendees’ Households only	116	7.12%	179	11.0%
Nonattendees in Attendees’ Social Networks and Households	165	10.1%	254	15.6%
**Total Possible Social Network and Household Reach**	**913**	**56.0%**	**1,236**	**75.8%**
Nonattendees not in Attendees’ Social Networks or Households	717	44.0%	394	24.2%

Data include *N* = 1,630 individuals who participated in 2016–2018 community survey.

Figures do not add to 100% due to rounding.

Similarly, in addition to the 464 study participants who attended a meeting before the community health fair, 593 (36%) participants had a geodesic distance of 1 from at least one of the attendees. The remaining 573 (35%) participants were more than 1 geodesic from an attendee. Compared with nonattendees who were not part of attendees’ social networks, nonattendees who were part of attendees’ social networks were more likely to be older age (mean age = 44.0 years versus 31.9 years, difference, 12.1 years, 95% CI, 10.2 to 13.9, *p* < 0.001), married (72.5% versus 41.9%, χ^2^ = 111.8, *p* < 0.001), and members of more community groups (1.02 groups versus 0.45 groups, difference, 0.57 groups, 95% CI, 0.47 to 0.67, *p* < 0.001) (S2 Table).

Comparing in-degree, out-degree, closeness centrality, and betweenness centrality, nonattendees (both for the community survey and the community health fair meetings) in attendees’ social networks were more socially integrated compared with nonattendees not in attendees’ social networks. Some alters were nominated by more than 1 attendee. For example, of the 533 participants with a geodesic distance of 1 from at least 1 attendee of a meeting before the community survey, 131 (25%) participants were named by 2 attendees, and 65 (12%) participants were named by 3 attendees.

Separately, in addition to the 264 study participants who attended a sensitization meeting before the community survey, 281 (17%) nonattendees lived in the same household as at least 1 attendee. Thus, information discussed during these meetings potentially reached 545 (33%) study participants. Compared with meeting attendees, these nonattendees living in attendees’ households were more likely to be younger age (36.1 years versus 45.1 years, difference, 8.98 years, 95% CI, 6.21 to 11.8, *p* < 0.001), men (56.9% versus 35.2%; χ^2^ = 25.8, *p* < 0.001), unmarried (46.6% versus 25.0%; χ^2^ = 27.6, *p* < 0.001), and members of fewer community groups (0.74 groups versus 1.32 groups, difference, 0.58 groups, 95% CI, 0.39 to 0.76, *p* < 0.001).

In addition to the 464 study participants who attended a meeting before the community health fair, 433 (27%) study participants who did not attend a meeting lived in attendees’ households, for a total household-level reach of 897 (55%) study participants. Compared with meeting attendees, these nonattendees living in attendees’ households were also more likely to be younger age (35.2 years versus 44.7 years, difference, 9.46 years, 95% CI, 7.37 to 11.6, *p* < 0.001), men (61.2% versus 30.0%; χ^2^ = 88.3, *p* < 0.001), unmarried (46.4% versus 30.2%; χ^2^ = 25.1, *p* < 0.001), and members of fewer community groups (0.71 groups versus 1.32 groups, difference, 0.61 groups, 95% CI, 0.46 to 0.75, *p* < 0.001). Comparing in-degree, out-degree, closeness centrality, and betweenness centrality (both for the community survey and the community health fair meetings), nonattendees in attendees’ households were less socially integrated than attendees.

Combined, 913 (56%) study participants either attended a sensitization meeting before the community survey or were in the social network or household of an attendee. Additionally, 1,236 (76%) study participants either attended a sensitization meeting before the community health fair or were in the social network or household of an attendee.

## Discussion

In this sociocentric social network cohort study from rural Uganda, we found that attendance at community sensitization meetings was correlated with a range of sociologically and economically meaningful characteristics. People who were older age and more socially integrated, women, and people who lived in close geographic proximity to the meeting location were more likely to attend community sensitization meetings. These findings suggest that the relationships and trust that are built through community sensitization activities may disproportionately extend to certain subgroups within the community. Investigators conducting health-related research studies in similar settings in rural, sub-Saharan Africa may need to engage in more targeted outreach if aiming to ensure representation by community members belonging to certain subgroups.

Study participants who attended community sensitization meetings were more likely to be older women. In Ankole culture, older-age individuals typically hold more power and make decisions within the family structure, which is consistent with the wisdom and respect accorded to older-age persons across some cultures [76]. As such, they may be more likely to attend meetings in order to gather information about upcoming study activities and relay information to others within their households, or they may be more likely to have discretionary time and availability to attend meetings held during a typical school- or workday. The greater representation of women in attendance at meetings held before the community health fair can potentially be explained by gender-unequal norms in Uganda that increase their likelihood of engaging in unpaid caregiving and domestic work [77], which may extend to attending meetings that provide information on research activities relevant to the health of their family members. Men’s attendance could also have been limited by idealized forms of masculinity that undermine engagement in health behaviors [78–80] or caregiving and domestic activities traditionally accorded to women [58,77], or by scheduling difficulties given their predominant work outside the village setting (e.g., as “bodaboda” [motorcycle taxis common to East Africa] drivers, casual laborers, and traders).

A number of other important variables were also correlated with attendance. Depending on the specification, proxies for economic status (e.g., household asset wealth quintile category) and social integration (e.g., being married, membership and participation in community groups) were also correlated with attendance. These estimates suggest that people who have greater economic status are less likely to attend community sensitization meetings, while people who are more socially integrated are more likely to attend. Community members who are less well-off economically may be more interested in attending meetings to learn about opportunities for free services, to receive study incentives, and to develop relationships with study staff members who may provide instrumental support in times of need. People who were more socially integrated may have been more likely to attend community sensitization meetings, either because they had more opportunities to hear about meeting times and locations or because they were simply more inclined to view participation favorably in the same way as participation in other community groups.

The extent to which these differentials manifested in differential spread of key information (e.g., about proposed study activities and their impacts on the community) is unknowable given our study design and data availability. In high-income countries, there are well-known disparities in either participation or opportunities to participate in clinical trials of therapeutics and other potentially beneficial health interventions such that racialized minorities, women, and older-age persons are underrepresented [81–84]. Human subjects research in resource-limited settings has often been characterized by exploitation [85,86]. However, given the importance of community sensitization meetings in ensuring awareness of and engagement in research activities, developing relationships and trust between participants and research institutions, and ensuring ethical good practice [6,7], it remains important to meet these goals in a way that ensures that all subgroups within a community have a voice in the conduct of human subjects research. We recognize the power relations between researchers and community members and the need to thoughtfully navigate these dynamics to prevent coercion within community engagement itself [87].

Of note, the sociocentric social network design of our cohort enabled us to determine that information conveyed during community sensitization meetings may have indirectly reached a broader portion of the population through social network or household connections. While attendees of the community sensitization meetings were more likely to be older, more socially integrated women, their social network ties were also more likely to be older and more socially integrated—but nonattendees who were indirectly exposed by virtue of living in the same household as an attendee were more likely to be younger, unmarried, and less socially integrated men. These findings suggest that information conveyed during meetings could be indirectly transmitted to community members from less well-represented sociodemographic subgroups.

The primary programmatic implication of our findings is that, while community sensitization efforts appear to reach a wide range of community members, more work is needed to understand how to better target younger, less socially integrated men [31]. It is possible that by holding such meetings at different times (e.g., on weekends or in the evenings) or in locations where younger men typically congregate (e.g., “bodaboda” stations), research teams may increase attendance at community sensitization meetings among men who work away from villages in rural settings. Such changes would need to be made in collaboration with local research staff to ensure appropriate remuneration in return for working outside of traditional working hours or to ensure they are able to manage their own caregiving and other responsibilities. A second implication of our findings is that, in the conduct of community sensitization meetings, researchers may wish to consider explicitly encouraging attendees to discuss the information conveyed, including details of upcoming study activities, with household members and other social ties. Future research might also assess the extent to which social networks facilitate the dissemination of key information conveyed during community sensitization meetings [88,89].

These findings may have relevance for the implementation of research and public health programs more broadly, particularly those that rely on community leader–led mobilization to support recruitment and resource distribution. Involving community leaders in mobilization efforts is key to community engagement, as it can diffuse power imbalances between researchers and community members, increase participation, and demonstrate respect for community structures [87]. However, programs need to identify the leaders and other “gatekeepers” within a given community, as well as those individuals that leaders and gatekeepers can reach through their networks. Considering access barriers for certain subgroups within the community, researchers and program administrators may wish to consider collaborating with community leaders and codeveloping targeted community mobilization plans to address blind spots. Initial meetings with community leaders can then lead to outreach with other community members. By integrating voices from multiple levels within a community structure, both during design and implementation, collaborative groups can increase buy-in, cultural humility, and community equity.

### Strengths and limitations

Strengths of this analysis include the whole-population design with full information about both attendees and nonattendees and the availability of data on objectively assessed (rather than self-reported) attendance. Yet interpretation of our findings is subject to certain limitations. First, while this analysis allowed us to examine demographic, health, economic, and social network characteristics associated with attendance at community sensitization meetings, we did not collect data on motivations for attendance. Qualitative studies may yield greater insight into the specific aspects of the meetings that motivate attendance. For example, individuals may attend because of genuine interest and investment in the research, perceived duty to the community, opportunities to socialize with friends, or the refreshments provided. Alternatively, as found by Dierickx and colleagues, some community members may not have attended any meetings due to either lack of awareness or time constraints. Future research in this area could be codesigned with community members. For example, focus groups could be used to elicit community members’ perceptions about why some individuals may or may not attend community sensitization meetings. This feedback could then be integrated into community surveys or the development of key informant interview guides.

Second, while our research teams sought to disseminate broadly all notices about upcoming community sensitization meetings, it was not an explicit aim to achieve a representative sample of community members in attendance. It is therefore possible that different aspects of the dissemination process, both structural and behavioral, led to skew in the distribution of community members attending. For example, VHT members and research assistants were disproportionately women, and older women are also more likely to attend church in this setting. Thus, because recruitment efforts were largely led by women or conducted in spaces commonly occupied by women, these efforts may have led to disproportionate attendance by women.

Third, our findings may not generalize beyond the study setting. However, the data are based on a whole-population study, and the study setting is broadly representative of rural regions in Eastern Africa. Although further study will be needed to replicate our findings, we expect that our findings will be relevant to other investigators conducting health-related research throughout sub-Saharan Africa. Finally, since we linked survey data collected between 2016 and 2018 to attendance data collected between 2018 and 2019, some of the demographic, health, economic, and social network correlates of attendance may have been out of date. For example, social ties that were present in 2016 to 2018 may have no longer been present by 2018 to 2019 (or, conversely, social ties that were not present in 2016 to 2018 could have formed by 2018 to 2019), which could have affected our estimates of the potential reach of information conveyed during community sensitization meetings.

## Conclusions

In this longitudinal population-based study, we found that women and people who were older age and more socially integrated were more likely to attend community sensitization meetings conducted in advance of research study activities in this rural region of southwestern Uganda. Individuals with lower socioeconomic status were also more likely to attend meetings. While significant proportions of the study population either attended a meeting or were indirectly exposed to a meeting through a social affiliation with a meeting attendee, our findings nonetheless raise some concerns that attendance at the meetings may be stratified along sociologically meaningful lines.

## Supporting information

S1 Checklist STROBE Checklist. (DOCX)

S1 Text Methods. (DOCX)

S1 Table Characteristics of study participants, stratified by attendance at sensitization meetings before the community survey, and attendees’ social network and household reach. (DOCX)

S2 Table Characteristics of study participants, stratified by attendance at sensitization meetings before the community health fair, and attendees’ social network and household reach. (DOCX)

## Abbreviations

ARR: adjusted relative risk
CAB: community advisory board
CI: confidence interval
IQR: interquartile range
LC1: local council level 1
RR: relative risk
SD: standard deviation
VHT: village health team
