# Supplementary material for: Correlates of attendance at community engagement meetings held in advance of bio-behavioral research studies: A longitudinal, sociocentric social network study in rural Uganda
Source: PLoS Med. 2021 Jul 16;18(7):e1003705. doi: 10.1371/journal.pmed.1003705 (PMC8323877; doi:10.1371/journal.pmed.1003705)
Supplement: S1 Text — (DOCX) [file pmed.1003705.s002.docx]

**S1 Text.** Methods

*Prespecified Analysis Plan*

No prospective protocol was published or registered for this observational study. However, we followed a clear analysis plan, as described in the methods section. The inclusion criteria for the study were established at the outset, in that a decision was made to include all eligible adults in the analysis, with no exclusions. The outcomes were prespecified at the outset. The statistical analyses were determined at the outset and were not changed. The various continuous and categorical specifications of the explanatory variables were prespecified, with minor changes, as described below.

Single-variable and multivariable Poisson regression models were fitted to estimate correlates of attendance at community sensitization meetings held before a community survey and a community health fair. The outcome variables were taken directly from attendance logs collected at the community sensitization meetings. No other community-wide sensitization meetings were held. Thus, there was no flexibility in how the outcome variables could have been specified, in that there were no other outcome variables that could have been chosen and no other ways the outcome variables could have been parameterized. The explanatory variables, including environmental, demographic, health, economic, and social network domains, were derived from data collected during a community survey conducted between 2016 and 2018. Using the sociocentric social network design of the study, we also calculated potential indirect exposure to information discussed during meetings. This extended reach of community sensitization meetings was calculated by summing attendees with non-attendees in attendees’ social networks and households.

During the peer review process, we were asked by a reviewer to calculate network characteristics of study participants, including in-degree, out-degree, closeness centrality, and betweenness centrality. These measures (median and interquartile range) were added into the Tables summarizing characteristics of study participants, stratified by attendance at meetings and social network and household reach of meeting attendees. In response to reviewer feedback, we updated the specification for the age variable in the Poisson regression models: while we had initially specified age as a categorical variable, at the request of a reviewer we instead specified age as a continuous variable.

As a result of this change to the age variable, the estimated regression coefficients derived from the Poisson regression models changed little, and the substantive conclusions of our analysis remained qualitatively similar. However, in the multivariable regression model estimating associations between attendance at a community sensitization meeting held before the community survey and various sociodemographic characteristics, the estimated association between attendance and female sex was no longer statistically significant, changing from an adjusted relative risk (ARR) of 1.41 (95% confidence interval [CI], 1.03-1.93, p=0.034) in the initial submission to an ARR of 1.38 (95% CI, 0.99-1.92, p=0.055) in the resubmitted manuscript.
