# Supplementary material for: Correlates of attendance at community engagement meetings held in advance of bio-behavioral research studies: A longitudinal, sociocentric social network study in rural Uganda
Source: PLoS Med. 2021 Jul 16;18(7):e1003705. doi: 10.1371/journal.pmed.1003705 (PMC8323877; doi:10.1371/journal.pmed.1003705)
Supplement: S1 Table — (DOCX) [file pmed.1003705.s003.docx]

**S1 Table.** Characteristics of study participants, stratified by attendance at sensitization meetings before the community survey, and attendees’ social network and household reach

|  | **Attendance at Any Community Survey Sensitization Meeting** | | | | | | | | | | | | | |
| --- | --- | --- | --- | --- | --- | --- | --- | --- | --- | --- | --- | --- | --- | --- |
|  | **Attendees** | | **Non-Attendees** | | | | | | | | **Combined Network Reach** | | | |
|  | **Attended Meeting**  (n=264, 16.2%) | | **In Attendee’s Social Network**  (n=533, 32.7%) | | **Not in Attendee’s Social Network**  (n=833, 51.1% | | **In Attendee’s Household**  (n=281, 17.2%) | | **Not in Attendee’s Household**  (n=1,085, 66.6%) | | **Attendee or in Attendees’ Social Network and/or Household**  (n=913, 56.0%) | | **Non-Attendee and Not in Attendees’ Social Network or Household**  (n=717, 44.0%) | |
|  | n | % | n | % | n | % | n | % | n | % | n | % | n | % |
| **Age Category:** | | | | | | |  |  |  |  |  |  |  |  |
| 18-25 years | 22 | 8.33% | 44 | 8.26% | 299 | 35.9% | 101 | 35.9% | 242 | 22.3% | 143 | 15.7% | 222 | 31.0% |
| 26-35 years | 59 | 22.4% | 127 | 23.8% | 219 | 26.3% | 64 | 22.8% | 282 | 26.0% | 212 | 23.2% | 193 | 26.9% |
| 36-45 years | 60 | 22.7% | 125 | 23.5% | 116 | 13.9% | 40 | 14.2% | 201 | 18.5% | 190 | 20.8% | 111 | 15.5% |
| 46-55 years | 62 | 23.5% | 118 | 22.1% | 84 | 10.1% | 38 | 13.5% | 164 | 15.1% | 181 | 19.8% | 83 | 11.6% |
| 56+ years | 56 | 21.2% | 111 | 20.8% | 104 | 12.5% | 35 | 12.5% | 180 | 16.6% | 173 | 19.0% | 98 | 13.7% |
| Missing | 5 | 1.89% | 8 | 1.50% | 11 | 1.32% | 3 | 1.07% | 16 | 1.47% | 14 | 1.53% | 10 | 1.39% |
| **Sex:** | | | | | | |  |  |  |  |  |  |  |  |
| Female | 171 | 64.8% | 308 | 57.8% | 432 | 51.9% | 121 | 43.1% | 619 | 57.1% | 526 | 57.6% | 385 | 53.7% |
| Male | 93 | 35.2% | 225 | 42.2% | 401 | 48.1% | 160 | 56.9% | 466 | 43.0% | 387 | 42.4% | 332 | 46.3% |
| **Married:** | | | | | | |  |  |  |  |  |  |  |  |
| Yes | 198 | 75.0% | 378 | 70.9% | 418 | 50.2% | 150 | 53.4% | 646 | 59.5% | 601 | 65.8% | 393 | 54.8% |
| No | 66 | 10.4% | 155 | 29.1% | 415 | 49.8% | 131 | 46.6% | 439 | 40.5% | 312 | 34.2% | 324 | 45.2% |
| **Education:** | | | | | | |  |  |  |  |  |  |  |  |
| Completed Primary School | 130 | 49.2% | 279 | 52.4% | 569 | 68.3% | 193 | 68.7% | 655 | 60.4% | 506 | 55.4% | 472 | 65.8% |
| Did Not Complete Primary School | 134 | 50.8% | 254 | 47.7% | 264 | 31.7% | 88 | 31.3% | 430 | 39.6% | 407 | 44.6% | 245 | 34.2% |
| **HIV Status:** | | | | | | |  |  |  |  |  |  |  |  |
| HIV Positive | 31 | 16.2% | 61 | 11.4% | 75 | 9.00% | 21 | 7.47% | 115 | 10.6% | 96 | 10.5% | 71 | 9.90% |
| HIV Negative | 233 | 88.3% | 472 | 88.6% | 758 | 91.0% | 260 | 92.5% | 970 | 89.4% | 817 | 89.5% | 646 | 90.1% |
| **Obese:** |  |  |  |  |  |  |  |  |  |  |  |  |  |  |
| Yes | 98 | 37.1% | 201 | 37.7% | 240 | 28.8% | 65 | 23.1% | 376 | 34.7% | 321 | 35.2% | 218 | 30.4% |
| No | 156 | 59.1% | 320 | 60.0% | 552 | 66.3% | 206 | 73.3%% | 666 | 61.4% | 564 | 61.8% | 464 | 64.7% |
| Missing | 10 | 3.79% | 12 | 2.25% | 41 | 4.92% | 10 | 3.56% | 43 | 3.96% | 28 | 3.07% | 35 | 4.88% |
| **Depression** |  |  |  |  |  |  |  |  |  |  |  |  |  |  |
| Median (IQR) | 1.40 | (1.20-1.77) | 1.40 | (1.20-1.73) | 1.33 | (1.33-1.67) | 1.33 | (1.13-1.60) | 1.40 | (1.20-1.67) | 1.40 | (1.20-1.73) | 1.33 | (1.13-1.67) |
| **Household Food Insecurity** | | | | | | |  |  |  |  |  |  |  |  |
| Food secure | 74 | 28.0% | 163 | 30.6% | 288 | 48.9% | 108 | 38.4% | 343 | 31.6% | 294 | 32.2% | 231 | 32.2% |
| Mild food insecurity | 34 | 12.9% | 71 | 13.3% | 102 | 11.2% | 33 | 11.7% | 140 | 12.9% | 113 | 12.4% | 94 | 13.1% |
| Moderate food insecurity | 109 | 41.3% | 215 | 40.3% | 296 | 21.4% | 97 | 34.5% | 414 | 38.2% | 360 | 39.4% | 260 | 36.3% |
| Severe food insecurity | 47 | 17.8% | 81 | 15.2% | 138 | 17.5% | 40 | 14.2% | 179 | 16.5% | 142 | 15.6% | 124 | 17.3% |
| Missing | 0 | 0.00% | 3 | 0.56% | 9 | 1.08% | 3 | 1.07% | 9 | 0.83% | 4 | 0.44% | 8 | 1.12% |
| **Household Water Insecurity** | | | | | | |  |  |  |  |  |  |  |  |
| Water secure | 130 | 49.2% | 257 | 48.2% | 407 | 48.9% | 142 | 50.5% | 522 | 48.1% | 448 | 49.1% | 346 | 48.3% |
| Mild water insecurity | 39 | 14.8% | 58 | 10.9% | 93 | 11.2% | 24 | 8.54% | 127 | 11.7% | 108 | 11.8% | 82 | 11.4% |
| Moderate food insecurity | 56 | 21.2% | 117 | 22.0% | 178 | 21.4% | 66 | 23.5% | 229 | 21.1% | 200 | 21.9% | 151 | 21.1% |
| Severe food insecurity | 39 | 14.8% | 97 | 18.2% | 146 | 17.5% | 45 | 16.0% | 198 | 18.3% | 152 | 16.7% | 130 | 18.1% |
| Missing | 0 | 0.00% | 4 | 0.75% | 9 | 1.08% | 4 | 1.42% | 9 | 0.83% | 5 | 0.55% | 8 | 1.12% |
| **Membership in Community Groups (No. of Groups)** | | | | | | |  |  |  |  |  |  |  |  |
| Median (range) | 1 | (0-9) | 1 | (0-6) | 0 | (0-6) | 1 | (0-6) | 1 | (0-6) | 1 | (0-9) | 0 | (0-6) |
| **Participation in Community Groups (No. of Groups)** | | | | | | |  |  |  |  |  |  |  |  |
| Median (range) | 1 | (0-8) | 1 | (0-6) | 0 | (0-5) | 0 | (0-6) | 0 | (0-6) | 1 | (0-8) | 0 | (0-5) |
| **Loneliness** | | | | | | |  |  |  |  |  |  |  |  |
| Median (IQR) | 3 | (3-5) | 3 | (3-4) | 3 | (3-5) | 3 | (3-4) | 3 | (3-5) | 3 | (3-4) | 3 | (3-5) |
| **Distance to Meetings in Village (km)** | | | | | | |  |  |  |  |  |  |  |  |
| Median (IQR) | 0.37 | (0.19-0.51) | 0.38 | (0.27-0.53) | 0.42 | (0.28-0.60) | 0.39 | (0.25-0.53) | 0.40 | (0.28-0.58) | 0.38 | (0.24-0.52) | 0.42 | (0.29-0.62) |
| **In-Degree**  Median (IQR) | 6 | (4-10.5) | 6 | (4-10) | 2 | (1-4) | 3 | (1-6) | 4 | (2-7) | 6 | (3-10) | 2 | (1-4) |
| **Out-Degree**  Median (IQR) | 6 | (4-8) | 6 | (4-8)_ | 5 | (3-6) | 5 | (4-7) | 5 | (4-7) | 6 | (4-7) | 5 | (3-6) |
| **Closeness Centrality** | | | | | | | | | | | | | | |
| Median (IQR) | 0.24 | (0.22-0.26) | 0.24 | (0.23-0.26) | 0.23 | (0.21-0.24) | 0.23 | (0.21-0.25) | 0.23 | (0.21-0.25) | 0.24 | (0.22-0.25) | 0.23 | (0.21-0.24) |
| **Betweenness Centrality** | | | | | | | | | | | | | | |
| Median (IQR) | 6038 | (2510—16415) | 6644 | (3004-14550) | 3699 | (160-4812) | 3148 | (318-8241) | 3396 | (778-8710) | 5642 | (1922-13147) | 1844 | (265-4870) |
| Abbreviations: IQR, interquartile range  ^a^ Figures do not add to 100% due to rounding | | | | | | |  |  |  |  |  | |  | |
