# Supplementary material for: Correlates of attendance at community engagement meetings held in advance of bio-behavioral research studies: A longitudinal, sociocentric social network study in rural Uganda
Source: PLoS Med. 2021 Jul 16;18(7):e1003705. doi: 10.1371/journal.pmed.1003705 (PMC8323877; doi:10.1371/journal.pmed.1003705)
Supplement: S2 Table — (DOCX) [file pmed.1003705.s004.docx]

**S2 Table.** Characteristics of study participants, stratified by attendance at sensitization meetings before the community health fair, and attendees’ social network and household reach

|  | **Attendance at Any Community Health Fair Sensitization Meeting** | | | | | | | | | | | | | |
| --- | --- | --- | --- | --- | --- | --- | --- | --- | --- | --- | --- | --- | --- | --- |
|  | **Attendees** | | **Non-Attendees** | | | | | | | | **Combined Network Reach** | | | |
|  | **Attended Meeting**  (n=464, 28.5%) | | **In Attendee’s Social Network**  (n=593, 36.4%) | | **Not in Attendee’s Social Network**  (n=573, 35.2% | | **In Attendee’s Household**  (n=433, 26.6%) | | **Not in Attendee’s Household**  (n=733, 45.0%) | | **Attendee or in Attendees’ Social Network and/or Household**  (n=1,236, 75.8%) | | **Non-Attendee and Not in Attendees’ Social Network or Household**  (n=394, 24.2%) | |
|  | n | % | n | % | n | % | n | % | n | % | n | % | n | % |
| **Age Category:** | | | | | | |  |  |  |  |  |  |  |  |
| 18-25 years | 44 | 9.48% | 65 | 11.0% | 256 | 44.7% | 166 | 38.3% | 155 | 21.2% | 234 | 18.9% | 131 | 33.3% |
| 26-35 years | 109 | 23.5% | 146 | 24.6% | 150 | 26.2% | 97 | 22.4% | 199 | 27.2% | 288 | 23.3% | 117 | 29.7% |
| 36-45 years | 100 | 21.6% | 122 | 20.6% | 79 | 13.8% | 59 | 13.6% | 142 | 19.4% | 233 | 18.9% | 68 | 17.3% |
| 46-55 years | 106 | 22.8% | 123 | 20.7% | 35 | 6.11% | 53 | 12.2% | 105 | 14.3% | 231 | 18.7% | 33 | 8.38% |
| 56+ years | 101 | 21.8% | 126 | 21.3% | 44 | 7.68% | 53 | 12.2% | 117 | 16.0% | 232 | 18.8% | 39 | 9.90% |
| Missing | 4 | 0.86% | 11 | 1.85% | 9 | 1.57% | 5 | 1.15% | 15 | 2.05% | 18 | 1.46% | 6 | 1.52% |
| **Sex:** | | | | | | |  |  |  |  |  |  |  |  |
| Female | 325 | 70.0% | 314 | 53.0% | 272 | 47.5% | 168 | 38.8% | 418 | 57.0% | 720 | 58.3% | 191 | 48.5% |
| Male | 139 | 30.0% | 279 | 47.1% | 301 | 52.5% | 265 | 61.2% | 315 | 43.0% | 516 | 41.8% | 203 | 51.5% |
| **Married:** | | | | | | |  |  |  |  |  |  |  |  |
| Yes | 324 | 69.8% | 430 | 72.5% | 240 | 41.9% | 232 | 53.6% | 438 | 59.8% | 789 | 63.8% | 205 | 52.0% |
| No | 140 | 30.2% | 163 | 27.5% | 333 | 58.1% | 201 | 46.4% | 295 | 40.3% | 447 | 36.2% | 189 | 48.0% |
| **Education:** | | | | | | |  |  |  |  |  |  |  |  |
| Completed Primary School | 239 | 51.5% | 323 | 54.4% | 416 | 72.6% | 300 | 69.3% | 439 | 59.9% | 712 | 57.6% | 266 | 67.5% |
| Did Not Complete Primary School | 225 | 48.5% | 270 | 45.5% | 157 | 27.4% | 133 | 30.7% | 294 | 40.1% | 524 | 42.4% | 128 | 32.5% |
| **HIV Status:** | | | | | | |  |  |  |  |  |  |  |  |
| HIV Positive | 58 | 12.5% | 62 | 10.5% | 47 | 8.20% | 29 | 6.70% | 80 | 10.9% | 127 | 10.3% | 40 | 10.2% |
| HIV Negative | 406 | 87.5% | 531 | 89.5% | 526 | 91.8% | 404 | 93.3% | 653 | 89.1% | 1,109 | 89.7% | 354 | 90.0% |
| **Obese:** |  |  |  |  |  |  |  |  |  |  |  |  |  |  |
| Yes | 183 | 39.4% | 217 | 36.6% | 139 | 24.3% | 101 | 23.3% | 255 | 34.8% | 433 | 35.0% | 106 | 26.9% |
| No | 258 | 55.6% | 364 | 61.4% | 406 | 70.9% | 320 | 73.9% | 450 | 61.4% | 762 | 61.7% | 266 | 67.5% |
| Missing | 23 | 4.96% | 12 | 2.02% | 28 | 4.89% | 12 | 2.77% | 28 | 3.82% | 41 | 3.32% | 22 | 5.58% |
| **Depression** |  |  |  |  |  |  |  |  |  |  |  |  |  |  |
| Median (IQR) | 1.40 | (1.20-1.73) | 1.40 | (1.20-1.67) | 1.33 | (1.13-1.60) | 1.27 | (1.13-1.60) | 1.40 | (1.20-1.67) | 1.40 | (1.20-1.67) | 1.33 | (1.17-1.67) |
| **Household Food Insecurity** | | | | | | |  |  |  |  |  |  |  |  |
| Food secure | 130 | 28.0% | 182 | 30.7% | 213 | 37.2% | 150 | 34.6% | 245 | 33.4% | 388 | 31.4% | 137 | 34.8% |
| Mild food insecurity | 50 | 10.8% | 82 | 13.8% | 75 | 13.1% | 58 | 13.4% | 99 | 13.5% | 153 | 12.4% | 54 | 13.7% |
| Moderate food insecurity | 199 | 42.9% | 238 | 40.1% | 183 | 31.9% | 165 | 38.1% | 256 | 34.9% | 493 | 39.9% | 127 | 32.2% |
| Severe food insecurity | 84 | 18.1% | 89 | 15.0% | 93 | 16.2% | 57 | 13.2% | 125 | 17.1% | 198 | 16.0% | 68 | 17.3% |
| Missing | 1 | 0.22% | 2 | 0.34% | 9 | 1.57% | 3 | 0.69% | 8 | 1.09% | 4 | 0.32% | 8 | 2.03% |
| **Household Water Insecurity** | | | | | | |  |  |  |  |  |  |  |  |
| Water secure | 220 | 47.4% | 282 | 47.6% | 292 | 51.0% | 215 | 49.7% | 359 | 49.0% | 588 | 47.6% | 206 | 52.3% |
| Mild water insecurity | 56 | 12.1% | 75 | 12.7% | 59 | 10.3% | 47 | 10.9% | 87 | 11.9% | 152 | 12.3% | 38 | 9.64% |
| Moderate food insecurity | 108 | 23.3% | 120 | 20.2% | 123 | 21.5% | 94 | 21.7% | 149 | 20.3% | 268 | 21.7% | 83 | 21.1% |
| Severe food insecurity | 79 | 17.0% | 114 | 19.2% | 89 | 15.5% | 73 | 16.9% | 130 | 17.7% | 223 | 18.0% | 59 | 15.0% |
| Missing | 1 | 0.22% | 2 | 0.34% | 10 | 1.75% | 4 | 0.92% | 8 | 1.09% | 5 | 0.40% | 8 | 2.03% |
| **Membership in Community Groups (No. of Groups)** | | | | | | |  |  |  |  |  |  |  |  |
| Median (range) | 1 | (0-9) | 1 | (0-6) | 0 | (0-4) | 0 | (0-6) | 1 | (0-6) | 1 | (0-9) | 0 | (0-4) |
| **Participation in Community Groups (No. of Groups)** | | | | | | |  |  |  |  |  |  |  |  |
| Median (range) | 1 | (0-8) | 1 | (0-6) | 0 | (0-4) | 0 | (0-5) | 0 | (0-6) | 0 | (0-8) | 0 | (0-4) |
| **Loneliness** | | | | | | |  |  |  |  |  |  |  |  |
| Median (IQR) | 3 | (3-5) | 3 | (3-4) | 3 | (3-5) | 3 | (3-4) | 3 | (3-5) | 3 | (3-4) | 3 | (3-5) |
| **Distance to Meetings in Village (km)** | | | | | | |  |  |  |  |  |  |  |  |
| Median (IQR) | 0.36 | (0.21-0.57) | 0.40 | (0.26-0.64) | 0.47 | (0.31-0.75) | 0.37 | (0.23-0.62) | 0.49 | (0.32-0.71) | 0.38 | (0.23-0.62) | 0.50 | (0.35-0.77) |
| **In-Degree**  Median (IQR) | 6 | (3-10) | 6 | (3-9) | 1 | (1-3) | 3 | (1-6) | 3 | (1-6) | 5 | (2-9) | 2 | (1-3) |
| **Out-Degree**  Median (IQR) | 6 | (4-8) | 6 | (4-7) | 4 | (3-6) | 5 | (4-7) | 5 | (3-7) | 6 | (4-7) | 4 | (3-6) |
| **Closeness Centrality** | | | | | | | | | | | | | | |
| Median (IQR) | 0.24 | (0.22-0.25) | 0.24 | (0.22-0.25) | 0.22 | (0.20-0.23) | 0.23 | (0.21-0.24) | 0.23 | (0.21-0.24) | 0.24 | (0.22-0.25) | 0.22 | (0.21-0.24) |
| **Betweenness Centrality** | | | | | | | | | | | | | | |
| Median (IQR) | 5920 | (2223-13718) | 5567 | (2625-12111) | 768 | (0.5-3244) | 2721 | (306-7504) | 3071 | (610-7886) | 4789 | (1593-11191) | 1057 | (85.7-3825) |
| Abbreviations: IQR, interquartile range  ^a^ Figures do not add to 100% due to rounding | | | | | | |  |  |  |  |  | |  | |
