# Supplementary material for: Correlates of attendance at community engagement meetings held in advance of bio-behavioral research studies: A longitudinal, sociocentric social network study in rural Uganda
Source: PLoS Med. 2021 Jul 16;18(7):e1003705. doi: 10.1371/journal.pmed.1003705 (PMC8323877; doi:10.1371/journal.pmed.1003705)
Supplement: Attachment — Submitted filename: kakuhikire.pdf [file pmed.1003705.s005.pdf]

This paper reports on correlates of attendance at community sensitisation meetings in rural Uganda.

I was asked for a statistical report and I interpret that to include all aspects of the design and conduct of the study.

Points of detail

Page 8 Is it possible to put the sum of money in context? How much does a bag of coffee cost (assuming they drink coffee in Uganda)? Or how much does a *bodaboda* driver earn a day? Incidentally that word may need explanation.

Page 11 It is a bit of a judgment call but I would have made the total in the first column 7 not 8.

Page 13 I imagine in an ideal world actual walking distance would be better but straight line is probably highly correlated.

Page 13 I think it might be helpful here to state how each variable is entered into the model. For instance age is in fact categorised not used as a continuous variable. Categorising an essentially continuous variable wastes information (Altman and Royston, 2006; Royston et al., 2006) and leads to models which are often implausible as they predict the effect remaining flat within categories and then jumping to a new value at the category boundary. So I wonder why age and other variables had to be categorised rather than used as continuous.

Page 14 The canonical way to get risk ratios is with a log–binomial model as the modified Poisson is only an approximation. Can the authors provide a justification?

Page 14 I do not think it is necessary to put handshake in quotes, the whole concept is quite widespread in diverse fields like Six Degrees of Kevin Bacon and Erdős numbers.

Page 17 I think the authors have been unduly influenced by arbitrary levels of statistical significance when they suggest that it was only attendance pre–health fair which is related to wealth. I would hardly think 0.59 could be seen as different from 0.57 in any meaningful way. If it is necessary to present the two sets of results separately then I think a narrative comparison of the sizes of the effects rather than they status above of below a cut–off would be more helpful.

Page 20 onwards We suddenly lose confidence intervals from here which is a shame.

In a couple of places the authors justify things by saying in accordance with local etiquette. The nearest equivalent to the scenario described here in my country would be the inclusion of patient and public involvement in trials and they are usually compensated for their attendance so I do not see the need to be apologetic here.

Point of more substance

I do not see a clear explanation offered for the decision to model the two types of meeting separately. I can make some guesses but the authors must know. It would give a clearer picture if the results could be integrated in some way but I would not want to offer explicit suggestions without knowing why the decision was taken to present them separately.

Summary

Mostly points of detail.

Michael Dewey

References

D G Altman and P Royston. The cost of dichotomising continuous variables. *British Medical Journal*, 332:1080, 2006.

P Royston, D G Altman, and W Sauerbrei. Dichotomizing continuous predictors in multiple regression: a bad idea. *Statistics in Medicine*, 25:127–141, 2006.
