# Supplementary material for: Correlates of attendance at community engagement meetings held in advance of bio-behavioral research studies: A longitudinal, sociocentric social network study in rural Uganda
Source: PLoS Med. 2021 Jul 16;18(7):e1003705. doi: 10.1371/journal.pmed.1003705 (PMC8323877; doi:10.1371/journal.pmed.1003705)
Supplement: Attachment — Submitted filename: commsensitiz_response-to-reviewers_plosmed_20210508.docx [file pmed.1003705.s006.docx]

**Response to Reviewers**

**Requests from the Academic Editor:**

**The one thing that is needed, from my perspective, is more information and context of social dynamics between the village health teams and other “gatekeepers” and the different segments of the community.**

**The implication is that this mechanism is one that allows people to participate and have voice, and is likely to be the one in which other health program or research access the community voice as well.**

**Would like to see some more discussion about implications and theory linking this practice to governance of studies and health programs. There are clearly voices in communities that have more or less access – does this need to be remedied, and if so, how?**

**What implications for research? Public health programs? What about studies and programs that rely on these networks to inform design? Are there community equity issues?**

RESPONSE: We thank the editor for these comments. We have added discussion around these important topics in the Discussion section.

“These findings may have relevance to the implementation of research and public health programs more broadly, particularly those that rely on community leader-led mobilization to support recruitment and resource distribution. Involving community leaders in mobilization efforts is key to community engagement, as it can diffuse power imbalances between researchers and community members, increase participation, and demonstrate respect for community structures [87]. However, programs need to identify the leaders and other “gatekeepers” within a given community, as well as those individuals that leaders and gatekeepers can reach through their networks. Considering access barriers for certain subgroups within the community, researchers and program administrators may wish to consider collaborating with community leaders and co-developing targeted community mobilization plans to address blind-spots. Initial meetings with community leaders can then lead to outreach with other community members. By integrating voices from multiple levels within a community structure, both during design and implementation, collaborative groups can increase buy-in, cultural humility, and community equity.” (page 31)

**Requests from the Editors:**

**1. Title: Please revise your title according to PLOS Medicine's style. Your title must be nondeclarative and not a question. It should begin with main concept if possible. "Effect of" should be used only if causality can be inferred, i.e., for an RCT. Please place the study design ("A randomized controlled trial," "A retrospective study," "A modelling study," etc.) in the subtitle (i.e., after a colon).**

RESPONSE: We have updated the title according to PLOS Medicine’s style. It now reads, “Correlates of attendance at community engagement meetings held in advance of bio-behavioral research studies: A longitudinal, sociocentric social network study in rural Uganda.”

**2. Abstract: Methods and Findings: Please include the study design, population and setting, number of participants, years during which the study took place, length of follow up, and main outcome measures. Please quantify the main results (with 95% CIs and p values). As the last sentence of the Abstract Methods and Findings section, please describe the main limitation(s) of the study's methodology.**

RESPONSE: The Methods and Findings section of the Abstract has been revised to include additional detail on the study design, population and setting, results, and limitations.

“**Methods and Findings**: Research assistants collected survey data from 1,630 adults participating in an ongoing sociocentric social network cohort study conducted in a rural region of southwestern Uganda. These community survey data, collected between 2016 and 2018, were linked to attendance logs from community sensitization meetings held in 2018 and 2019 before the subsequent community survey and community health fair. Of all participants, 264 (16%) attended a community sensitization meeting before the community survey, 464 (28%) attended a meeting before the community health fair, 558 (34%) attended a meeting before either study activity (survey or health fair), and 170 (10%) attended a meeting before both study activities (survey and health fair). Using multivariable Poisson regression models, we estimated correlates of attendance at community sensitization meetings. Attendance was more likely among study participants who were women (adjusted relative risk [ARR]_survey_=1.38, 95% confidence interval [CI], 0.99-1.92, p=0.55; ARR_health fair_=1.71, 95% CI, 1.32-2.21, p<0.001), older age (ARR_survey_=1.02 per year, 95% CI, 1.01-1.02, p<0.001; ARR_health fair_=1.02 per year, 95% CI, 1.01-1.02, p<0.001), married (AR _survey_=1.74, 95% CI, 1.29-2.35, p<0.001; ARR_health fair_=1.41, 95% CI, 1.13-1.76, p=0.002), and members of more community groups (ARR_survey_=1.26 per group, 95% CI, 1.10-1.44, p=0.001; ARR_health fair_=1.26 per group, 95% CI, 1.12-1.43, p<0.001). Attendance was less likely among study participants who lived farther from meeting locations (ARR_survey_=0.54 per kilometer, 95% CI, 0.30-0.97, p=0.041; ARR_health fair_=0.57 per kilometer, 95% CI, 0.38-0.86, p=0.007). Leveraging the cohort’s sociocentric design, social network analyses suggested that information conveyed during community sensitization meetings could reach a broader group of potential study participants through attendees’ social network and household connections. Study limitations include: lack of detailed data on reasons for attendance/non-attendance at community sensitization meetings; achieving a representative sample of community members was not an explicit aim of the study; and generalizability may not extend beyond this study setting.” (page 3)

**3. Abstract: Conclusions: Please include a sentence interpreting the study’s key findings; the phrase "In this study, we observed ..." may be useful.**

RESPONSE: We have added two sentences to the beginning of the Conclusions section of the Abstract to summarize the study’s key findings.

“**Conclusions:** In this longitudinal, sociocentric social network study conducted in rural Uganda, we observed that older age, female sex, being married, membership in more community groups, and geographical proximity to meeting locations were correlated with attendance at community sensitization meetings held in advance of bio-behavioral research activities. Information conveyed during meetings could have reached a broader portion of the population through attendees’ social network and household connections.” (page 3)

**4. Author Summary: At this stage, we ask that you include a short, non-technical Author Summary of your research to make findings accessible to a wide audience that includes both scientists and non-scientists. The Author Summary should immediately follow the Abstract in your revised manuscript. This text is subject to editorial change and should be distinct from the scientific abstract. Please see our author guidelines for more information:** [**https://secure-web.cisco.com/1-oYP1E7GzN3E96QNqsD35jYUx9aNmjza4Y-u1-PHacqh4_VodjO4K7i8KvyE81VeURgVaHFDQtdNMlZSigWD_5pynvH8MlpHBg1YVp0Tzi_hU7UqP2cYDVjakttRJ-s3D3ymtT0GPFZDWK5_9z6TLQ_GaytVyAY2NYY1KPEmaD3eM9CfknQJYd9EltJtXwknIC-n3Nnv9lZVIfcOCAH6K_G61wsItmqsXWkfNyva8Qai4O4PofyhvvsY_iDz1lGy/https%3A%2F%_2Fjournals_.plos.org%2Fplosmedicine%2Fs%2Frevising-your-manuscript%23loc-author-summary**](https://secure-web.cisco.com/1-oYP1E7GzN3E96QNqsD35jYUx9aNmjza4Y-u1-PHacqh4_VOdjO4K7i8KVyE81VeURgVaHFDQtdNMlZSigWD_5pynvH8MlpHBg1YVp0TZi_hU7UqP2cYDVjakttRJ-s3D3ymtT0GPFZDWK5_9z6TLQ_GAytVyAY2NYY1KPEmaD3eM9CfknQJYd9EltJtXwknIC-n3Nnv9lZVIfcOCAH6K_G61wsItmqsXWkfNyva8Qai4O4POfyhvvsY_iDz1lGy/https%3A%2F%2Fjournals.plos.org%2Fplosmedicine%2Fs%2Frevising-your-manuscript%23loc-author-summary)

RESPONSE: We now include a non-technical Author Summary on pages 4-5.

**“Why was this study done?**

- Community engagement is central to the conduct of health-related research studies as a means of developing trust, increasing awareness of and engagement with research procedures, and safeguarding ethical good practice.
- Community sensitization meetings are often held in advance of global health research activities to build community awareness of key scientific and research concepts and to create opportunities for collaboration and feedback.
- Since successful research implementation requires buy-in from a range of stakeholders, fulfillment of community sensitization meeting aims requires widespread attendance and, potentially, subsequent dissemination of information to community members not in attendance.

**What did the researchers do and find?**

- We conducted a longitudinal, sociocentric social network study with 1,630 adults in a rural region of southwestern Uganda to understand the demographic, health, economic, and social network correlates of attendance at community sensitization meetings held before two research study activities.
- Attendance at community sensitization meetings was more likely among study participants who were older age, women, married, members of more community groups, and living in closer geographical proximity to the meeting locations.
- Non-attendees living in the households of meeting attendees were more likely (compared with meeting attendees) to be younger age, men, unmarried, and members of fewer community groups.

**What do these findings mean?**

- These findings suggest that information dissemination, relationships and trust achieved through the use of community sensitization meetings held in advance of research studies may disproportionately extend to certain sociodemographic subgroups.
- However, information conveyed during community sensitization meetings may reach a broader sample of the population via informal transmission through attendees’ social network and household connections.
- Future community sensitization efforts should aim to better target members of underrepresented groups.”

**5. Methods: Please ensure that the study is reported according to the STROBE guideline, and include the completed STROBE checklist as Supporting Information. Please add the following statement, or similar, to the Methods: "This study is reported as per the Strengthening the Reporting of Observational Studies in Epidemiology (STROBE) guideline (S1 Checklist)."**

**The STROBE guideline can be found here:** [**http://secure-web.cisco.com/1CeylT1nw01w146bdMajl1Ii_wX77yfLe5ha6SaFsDAZzkIisLkDY8Le08VmBhI6hgY3yGIhPZFe294Fzki7eAzBKdOjELYcnbKYt9qKu5IRP9UFGJvGHNT7CNNnpbeMlspwuJF-XSoAywvaY_JL63XEUiYD6aRUbDHABtFjgO8QE-Nw3GXarNLJ6zS8PfCcbN_V_g0mE75mKjHwXMyBY2ILDMujXNgxIzFdOOUb8gfm4mHwiAwVc7rcIu-oL-1bO/http%3A%2F%2Fwww.equator-network.org%2Freporting-guidelines%2Fstrobe%2F**](http://secure-web.cisco.com/1CeylT1nw01w146bdMajl1Ii_wX77yfLe5ha6SaFsDAZzkIisLkDY8Le08VmBhI6hgY3yGIhPZFe294Fzki7eAzBKdOjELYcnbKYt9qKu5IRP9UFGJvGHNT7CNNnpbeMlspwuJF-XSoAywvaY_JL63XEUiYD6aRUbDHABtFjgO8QE-Nw3GXarNLJ6zS8PfCcbN_V_g0mE75mKjHwXMyBY2ILDMujXNgxIzFdOOUb8gfm4mHwiAwVc7rcIu-oL-1bO/http%3A%2F%2Fwww.equator-network.org%2Freporting-guidelines%2Fstrobe%2F)

**When completing the checklist, please use section and paragraph numbers, rather than page numbers.**

RESPONSE: The STROBE Checklist is now included as S1 Checklist. All items have been completed.

**6. Methods: Did your study have a prospective protocol or analysis plan? Please state this (either way) early in the Methods section.**

RESPONSE: We did not have a prospective protocol. At the beginning of the Data Analysis section of the Methods, we state that while we did not have a registered study protocol, we followed a clear analysis plan.

“The analysis was not pre-registered, but we followed a prespecified analysis plan and tracked deviations that resulted from peer review (S1 Text).” (page 17)

**a) If a prospective analysis plan (from your funding proposal, IRB or other ethics committee submission, study protocol, or other planning document written before analyzing the data) was used in designing the study, please include the relevant prospectively written document with your revised manuscript as a Supporting Information file to be published alongside your study, and cite it in the Methods section. A legend for this file should be included at the end of your manuscript.**

RESPONSE: At the beginning of the Data Analysis section of the Methods, we provide a reference to our analysis plan (S1 Text). This file is cited as a legend at the end of the manuscript.

**b) If no such document exists, please make sure that the Methods section transparently describes when analyses were planned, and when/why any data-driven changes to analyses took place.**

RESPONSE: We have included the data analysis plan (Methods; S1 Text) as a Supporting information file. The text of the S1 Text reads:

“No prospective protocol was published or registered for this observational study. However, we followed a clear analysis plan, as described in the methods section. The inclusion criteria for the study were established at the outset, in that a decision was made to include all eligible adults in the analysis, with no exclusions. The outcomes were prespecified at the outset. The statistical analyses were determined at the outset and were not changed. The various continuous and categorical specifications of the explanatory variables were prespecified, with minor changes, as described below.

Single-variable and multivariable Poisson regression models were fitted to estimate correlates of attendance at community sensitization meetings held before a community survey and a community health fair. The outcome variables were taken directly from attendance logs collected at the community sensitization meetings. No other community-wide sensitization meetings were held. Thus, there was no flexibility in how the outcome variables could have been specified, in that there were no other outcome variables that could have been chosen and no other ways the outcome variables could have been parameterized. The explanatory variables, including environmental, demographic, health, economic, and social network domains, were derived from data collected during a community survey conducted between 2016 and 2018. Using the sociocentric social network design of the study, we also calculated potential indirect exposure to information discussed during meetings. This extended reach of community sensitization meetings was calculated by summing attendees with non-attendees in attendees’ social networks and households.

During the peer review process, we were asked by a reviewer to calculate network characteristics of study participants, including in-degree, out-degree, closeness centrality, and betweenness centrality. These measures (median and interquartile range) were added into the Tables summarizing characteristics of study participants, stratified by attendance at meetings and social network and household reach of meeting attendees. In response to reviewer feedback, we updated the specification for the age variable in the Poisson regression models: while we had initially specified age as a categorical variable, at the request of a reviewer we instead specified age as a continuous variable.

As a result of this change to the age variable, the estimated regression coefficients derived from the Poisson regression models changed little, and the substantive conclusions of our analysis remained qualitatively similar. However, in the multivariable regression model estimating associations between attendance at a community sensitization meeting held before the community survey and various sociodemographic characteristics, the estimated association between attendance and female sex was no longer statistically significant, changing from an adjusted relative risk (ARR) of 1.41 (95% confidence interval [CI], 1.03-1.93; P=0.034) in the initial submission to an ARR of 1.38 (95% CI, 0.99-1.92; P=0.055) in the resubmitted manuscript.”

**c) In either case, changes in the analysis-- including those made in response to peer review comments-- should be identified as such in the Methods section of the paper, with rationale.**

RESPONSE: As described above, in S1 Text, we note that we updated the Poisson regression models so that age was included as a continuous variable rather than as a categorical variable. We also mention that we calculated individual network characteristics of the study sample (in-degree, out-degree, closeness centrality, betweenness centrality) and included these measures in the descriptive statistics tables (Table 2, S1 Table, S2 Table)

**7. Methods: Please clarify the years during which the study took place.**

RESPONSE: Near the beginning of the Methods, we state, “The analysis was conducted using data collected between 2016 and 2019 as part of a longitudinal, sociocentric social network study in rural Uganda [54].” (page 9)

**8. Methods: Is it a potential limitation of the study that the linked survey data were from 2016-2018 while the attendance information was from 2018-2020 (potentially out of date social network, health, demographic data)?**

RESPONSE: We agree that this is a potential limitation, although we also believe there are advantages (i.e., because the data were collected prospectively, the predictors temporally precede the outcomes). Nonetheless, we have added this as an additional limitation in the Discussion section.

“Finally, since we linked survey data collected between 2016 and 2018 to attendance data collected between 2018 and 2019, some of the demographic, health, economic, and social network correlates of attendance may have been out of date. For example, social ties that were present in 2016-18 may have no longer been present by 2018-19 (or, conversely, social ties that were not present in 2016-18 could have formed by 2018-19), which could have affected our estimates of the potential reach of information conveyed during community sensitization meetings.” (page 33)

**9. Table 3: Please note the variables used in adjustment in the legend.**

RESPONSE: At the bottom of Table 3, we now list the variables used in adjustment.

“The adjusted models include each of the variables listed in the table row headers: age, sex, marital status, education, HIV status, obesity, depression symptom severity, household food insecurity, household water insecurity, household asset wealth, membership in community groups, participation in community groups, loneliness, and straight-line distance to the meeting corresponding to participant’s village of residence.”

**10. References: Please use the "Vancouver" style for reference formatting (including NLM journal name abbreviations), and see our website for other reference guidelines** [**https://secure-web.cisco.com/1FUgjjR_80z__rUF2CqVfmlG_iY1OwqPYw4bSUV45nqwA2lK9S8fyzLIVpa4dTH9AuIt3RwFCAOAK6SkFD-ZHct1Ci0nbcrHuaUyweNmK8BtueAH53y_uBgIPhn-EipMWAxA-L5VGntbN0ch_c6GrmgvadPNQmvQY0SRRKEZ5sz6QlzNudsE5vrrw-TTTNz5zz5fUadY2Mxp2oZZM-YqKwZOwU-HOnYsdBEkCMmwTf8A625SCMfHqCaGm2RQJdRGP/https%3A%2F%2Fjournals.plos.org%2Fplosmedicine%2Fs%2Fsubmission-guidelines%23loc-references**](https://secure-web.cisco.com/1FUgjjR_80z__rUF2CqVfmlG_iY1OwqPYw4bSUV45nqwA2lK9S8fyzLIVpa4dTH9AuIt3RwFCAOAK6SkFD-ZHct1Ci0nbcrHuaUyweNmK8BtueAH53y_uBgIPhn-EipMWAxA-L5VGntbN0ch_c6GrmgvadPNQmvQY0SRRKEZ5sz6QlzNudsE5vrrw-TTTNz5zz5fUadY2Mxp2oZZM-YqKwZOwU-HOnYsdBEkCMmwTf8A625SCMfHqCaGm2RQJdRGP/https%3A%2F%2Fjournals.plos.org%2Fplosmedicine%2Fs%2Fsubmission-guidelines%23loc-references)

RESPONSE: The references have been updated to reflect these formatting guidelines.

**Comments from the reviewers:**

**Reviewer #1:**

**This paper reports on correlates of attendance at community sensitization meetings in rural Uganda.**

**I was asked for a statistical report and I interpret that to include all aspects of the design and conduct of the study.**

**Points of detail:**

**1. Page 8: Is it possible to put the sum of money in context? How much does a bag of coffee cost (assuming they drink coffee in Uganda)? Or how much does a *bodaboda* driver earn a day? Incidentally that word may need explanation.**

RESPONSE: The revised text now contextualizes the study participation incentive.

“In return for their assistance, these stakeholders were provided with 10,000 Ugandan Shillings (approximately 2.70 USD – the value of 2 kg sugar – given the exchange rate at the time the study was conducted).” (page 10)

In the Discussion, we now define *bodaboda* the first time it is mentioned.

“…or by scheduling difficulties given their predominant work outside the village setting (e.g., as *bodaboda* [motorcycle taxis common to East Africa] drivers, casual laborers, and traders).” (page 29)

**2. Page 11: It is a bit of a judgment call, but I would have made the total in the first column 7 not 8.**

RESPONSE: While we included the attendance log from a community sensitization meeting held in advance of the community survey in Rwembogo, we forgot to include a 1 next to Rwembogo in the first column of Table 1. We have updated the table accordingly. Table 1 now includes the correct totals for the number of meetings held before the different study activities that were *included* in the analysis: 8 before the community survey, 16 before the community health fair, and 24 overall. (page 14)

**3. Page 13: I imagine in an ideal world actual walking distance would be better but straight line is probably highly correlated.**

RESPONSE: We agree that actual walking distance would be a more accurate measure of distance from participants’ houses to the meeting locations. However, roads within the parish are not all well-marked, and many participants will hike across properties and through their *matooke* (local plantain) gardens to get to the meetings. As such, we were unable to measure either walking distance or driving distance, and therefore defaulted to straight-line distance as the best proxy given the constraints of the parish geography.

**4.** **Page 13: I think it might be helpful here to state how each variable is entered into the model. For instance, age is in fact categorized not used as a continuous variable. Categorizing an essentially continuous variable wastes information (Altman and Royston, 2006; Royston et al, 2006) and leads to models which are often implausible as they predict the effect remaining flat within categories and then jumping to a new value at the category boundary. So, I wonder why age and other variables had to be categorized rather than used as continuous.**

RESPONSE: In the “Explanatory Variables” subsection of the Methods section, we now state how each variable was entered into the model. We additionally updated our Poisson regression models to specify age as a continuous variable rather than as a categorical variable. Table 3 now includes the updated risk ratios, confidence intervals, and p-values. When age is specified as a continuous variable, female sex no longer has a statistically significant association with “attendance at a community sensitization meeting held in advance of the community survey”, but the association between female sex and “attendance at a community sensitization meeting held in advance of the community health fair” remains statistically significant.

**5. Page 14: The canonical way to get risk ratios is with a log-binomial model as the modified Poisson is only an approximation. Can the authors provide a justification?**

RESPONSE: We agree with the reviewer that log-binomial regression would be the conventional means for estimating the relative risk ratios. However, we recognize that on occasion the estimated confidence intervals from log-binomial regression models can be excessively narrow (Skov et al. Int J Epidemiol 1998;29:91-5; Ma & Wong Int J Epidemiol 1999;28:175). We did attempt to fit log-binomial regression models to the data, but the regression model failed to converge for the model predicting “attendance at a community sensitization meeting held in advance of the community health fair” as the dependent variable. (The model for “attendance at a community sensitization meeting held in advance of the community survey” did converge.) We provide a table of the log-binomial estimates below, excluding the models that didn’t converge, so that the reviewer can compare the estimates side by side with those reported in Table 3 of the manuscript.

**Reviewer Table 1.** Unadjusted and multivariable log-binomial regression models estimating correlates of attendance at community sensitization meetings.

*Note: Estimates correspond to the Poisson regression estimates in Table 3 of the manuscript. The multivariable log-binomial model for correlates of attendance at a meeting held in advance of the community health fair is excluded since the model did not converge.*

|  | **Attendance at Community Sensitization Meeting Held in Advance of Community Survey** | | | | **Attendance at Community Sensitization Meeting Held in Advance of Community Health Fair** | |
| --- | --- | --- | --- | --- | --- | --- |
|  | **RR**  **(95% CI)** | ***p*-value** | **ARR**  **(95% CI)** | ***p*-value** | **RR**  **(95% CI)** | ***p*-value** |
| **Age** | 1.01  (1.01-1.02) | <0.001 | 1.02  (1.01-1.02) | <0.001 | 1.01  (1.01-1.02) | <0.001 |
| **Female** | 1.45  (1.10-1.91) | 0.009 | 1.34  (0.99-1.82) | 0.060 | 1.85  (1.41-2.41) | <0.001 |
| **Married** | 1.79  (1.31-2.46) | <0.001 | 1.65  (1.16-2.34) | 0.006 | 1.48  (1.20-1.83) | <0.001 |
| **Completed Primary School** | 0.68  (0.53-0.88) | 0.003 | 0.97  (0.79-1.18) | 0.735 | 0.71  (0.58-0.86) | <0.001 |
| **HIV Positive** | 1.13  (0.66-1.94) | 0.662 | 1.14  (0.60-2.15) | 0.692 | 1.25  (0.93-1.69) | 0.144 |
| **Obese** | 1.20  (0.92-1.56) | 0.175 | 0.93  (0.66-1.31) | 0.666 | 1.35  (1.11-1.65) | 0.003 |
| **Depression Symptom Severity** | 1.38  (1.13-1.68) | 0.002 | 1.27  (0.99-1.65) | 0.064 | 1.25  (1.08-1.44) | 0.003 |
| **Household Food Insecurity:**  Food Secure (*reference*) |  |  |  |  |  |  |
| Mild Food Insecurity | 1.16  (0.79-1.70) | 0.451 | 0.91  (0.55-1.50) | 0.717 | 0.98  (0.70-1.35) | 0.881 |
| Moderate Food Insecurity | 1.28  (0.73-2.25) | 0.381 | 1.02  (0.56-1.87) | 0.946 | 1.30  (0.99-1.70) | 0.060 |
| Severe Food Insecurity | 1.22  (0.76-1.95) | 0.402 | 0.93  (0.62-1.39) | 0.726 | 1.28  (1.02-1.59) | 0.030 |
| **Household Water Insecurity:**  Water Secure (*reference*) |  |  |  |  |  |  |
| Mild Water Insecurity | 1.30  (0.99-1.70) | 0.056 | 1.14  (0.93-1.41) | 0.204 | 1.06  (0.89-1.26) | 0.485 |
| Moderate Water Insecurity | 1.07  (0.70-1.64) | 0.754 | 0.89  (0.60-1.33) | 0.576 | 1.11  (0.95-1.30) | 0.199 |
| Severe Water Insecurity | 0.91  (0.63-1.31) | 0.594 | 0.78  (0.61-1.00) | 0.048 | 1.01  (0.81-1.26) | 0.923 |
| **Household Asset Wealth Quintile**  1st (Poorest, *reference*) |  |  |  |  |  |  |
| 2nd | 1.17  (0.79-1.71) | 0.434 | 1.09  (0.64-1.87) | 0.741 | 1.01  (0.86-1.19) | 0.906 |
| 3rd | 1.09  (0.91-1.30) | 0.357 | 1.09  (0.87-1.38) | 0.448 | 1.04  (0.96-1.12) | 0.320 |
| 4th | 1.00  (0.85-1.16) | 0.957 | 0.90  (0.61-1.31) | 0.573 | 0.92  (0.76-1.11) | 0.393 |
| 5th (Least poor) | 0.62  (0.37-1.05) | 0.079 | 0.59  (0.33-1.08) | 0.088 | 0.58  (0.41-0.81) | 0.001 |
| **Membership in Community Groups (No.)** | convergence not achieved | | 1.30  (1.10-1.54) | 0.002 | 1.27  (1.23-1.31) | <0.001 |
| **Participation in Community Groups (No.)** | convergence not achieved | | 1.02  (0.83-1.26) | 0.864 | convergence not achieved | |
| **Loneliness** | 1.01  (0.93-1.10) | 0.796 | 0.99  (0.90-1.07) | 0.747 | 1.01  (0.98-1.05) | 0.376 |
| **Distance to Meetings in Village (km)** | 0.51  (0.27-0.95) | 0.034 | 0.52  (0.27-1.01) | 0.053 | 0.55  (0.38-0.79) | 0.001 |
| **Constant** |  |  | 0.04  (0.02-0.10) | <0.001 |  |  |
| **Observations** |  | | 1,297 | |  | |
| Abbreviations: RR, relative risk; ARR, adjusted relative risk; CI, confidence interval  The adjusted models include each of the variables listed in the table row headers: age, sex, marital status, education, HIV status, obesity, depression symptom severity, household food insecurity, household water insecurity, household asset wealth, membership in community groups, participation in community groups , loneliness, and straight-line distance to the meeting corresponding to participant’s village of residence | | | | | | |

**6. Page 14: I do not think it is necessary to put handshake in quotes, the whole concept is quite widespread in diverse fields like Six Degrees of Kevin Bacon and Erdos numbers.**

RESPONSE: We have removed the quotation marks around handshake both times it appears in the text.

**7.** **Page 17: I think the authors have been unduly influenced by arbitrary levels of statistical significance when they suggest that it was only attendance pre-health fair which is related to wealth. I would hardly think 0.59 could be seen as different from 0.57 in any meaningful way. If it is necessary to present the two sets of results separately then I think a narrative comparison of the sizes of the effects rather than the status above or below a cut-off would be more helpful.**

RESPONSE: In the revised text, we now comment on these differing associations. We agree with the reviewer that the ARR of 0.57 is substantively similar to the ARR of 0.59 (now shifted to 0.61, with the specification of age as a continuous variable). The revised text now reads:

“There were slight differences in some of the estimated associations, in terms of convention thresholds of statistical significance. For example, women were more likely to have attended a community sensitization meeting before the health fair (ARR=1.71, 95% CI, 1.32-2.21, p<0.001), but not more likely to have attended a community sensitization meeting held before the community survey (ARR=1.38, 95% CI, 0.99-1.92, p=0.055). Furthermore, compared with individuals from the poorest household asset wealth quintile category, the least poor individuals were less likely to have attended a community sensitization meeting before the health fair (ARR=0.57, 95% CI, 0.41-0.80, p=0.001); the least poor individuals were similarly less likely to have attended a community sensitization meeting before the community survey, but the estimated association was not statistically significant (ARR=0.61, 95% CI, 0.34-1.10, p=0.10).” (page 22)

**8.** **Page 20 and onwards: We suddenly lose confidence intervals from here which is a shame.**

RESPONSE: We now include confidence intervals throughout the Results.

**9. In a couple places the authors justify things by saying in accordance with local etiquette. The nearest equivalent to the scenario described here in my country would be the inclusion of patient and public involvement in trials and they are usually compensated for their attendance, so I do not see the need to be apologetic here.**

RESPONSE: We previously justified compensating study participants for their time by including the phrase “consistent with local etiquette”. We have removed this phase from the text in both instances.

**Points of more substance:**

**10.** **I do not see a clear explanation offered for the decision to model the two types of meeting separately. I can make some guesses, but the authors must know. It would give a clearer picture if the results could be integrated in some way, but I would not want to offer explicit suggestions without knowing why the decision was taken to present them separately.**

RESPONSE: The revised text now explicitly describes our justification for modeling the two outcomes separately:

“We fitted separate regression models for the two dichotomous attendance variables since we hypothesized that participants from different sociodemographic subgroups might be interested in attending meetings before the study activities for different reasons. For example, the community health fair provided a clear service (i.e., disease screening/testing and referral for treatment), so individuals with health concerns or individuals whose family members had health problems might be more likely to attend.” (page 17)

For the reviewer’s perusal, we fitted multivariable Poisson regression models specifying “attendance at a community sensitization meeting held before either study activity (community survey and/or community health fair)” as the dependent variable, with the same set of covariates as shown in Table 3. This supplementary analysis is included below.

**Reviewer Table 2.** Unadjusted and multivariable Poisson regression models estimating correlates of attendance at any community sensitization meetings.

*Note: Estimates correspond to the Poisson regression estimates in Table 3 of the manuscript.*

|  | **Attendance at Community Sensitization Meeting Held in Advance of Either Study Activity (Community Survey and/or Community Health Fair)** | | | |
| --- | --- | --- | --- | --- |
|  | **RR (95% CI)** | ***p*-value** | **ARR (95% CI)** | ***p*-value** |
| **Age** | 1.01 (1.01-1.02) | <0.001 | 1.02 (1.01-1.02) | <0.001 |
| **Female** | 1.67 (1.35-2.07) | <0.001 | 1.61 (1.30-1.98) | <0.001 |
| **Married** | 1.51 (1.22-1.87) | <0.001 | 1.48 (1.18-1.86) | 0.001 |
| **Completed Primary School** | 0.68 (0.57-0.80) | <0.001 | 0.99 (0.86-1.15) | 0.930 |
| **HIV Positive** | 1.22 (0.95-1.55) | 0.119 | 1.13 (0.90-1.41) | 0.312 |
| **Obese** | 1.20 (0.99-1.45) | 0.062 | 0.91 (0.78-1.06) | 0.232 |
| **Depression Symptom Severity** | 1.27 (1.10-1.46) | 0.001 | 1.07 (0.91-1.26) | 0.403 |
| **Household Food Insecurity:**  Food Secure (*reference*) |  |  |  |  |
| Mild Food Insecurity | 0.94 (0.76-1.16) | 0.576 | 0.81 (0.63-1.04) | 0.094 |
| Moderate Food Insecurity | 1.27 (0.96-1.68) | 0.099 | 1.00 (0.74-1.34) | 0.993 |
| Severe Food Insecurity | 1.25 (1.02-1.55) | 0.034 | 0.95 (0.79-1.14) | 0.561 |
| **Household Water Insecurity:**  Water Secure (*reference*) |  |  |  |  |
| Mild Water Insecurity | 1.17 (0.97-1.41) | 0.107 | 1.02 (0.89-1.17) | 0.763 |
| Moderate Water Insecurity | 1.13 (0.97-1.31) | 0.106 | 1.02 (0.93-1.12) | 0.711 |
| Severe Water Insecurity | 1.07 (0.87-1.32) | 0.531 | 0.95 (0.82-1.11) | 0.520 |
| **Household Asset Wealth Quintile**  1st (Poorest, *reference*) |  |  |  |  |
| 2nd | 0.97 (0.81-1.16) | 0.733 | 0.92 (0.76-1.12) | 0.401 |
| 3rd | 0.94 (0.87-1.01) | 0.095 | 0.89 (0.82-0.97) | 0.008 |
| 4th | 0.82 (0.72-0.93) | 0.003 | 0.74 (0.64-0.86) | <0.001 |
| 5th (Least poor) | 0.53 (0.42-0.68) | <0.001 | 0.54 (0.40-0.72) | <0.001 |
| **Membership in Community Groups (No.)** | 1.29 (1.20-1.38) | <0.001 | 1.22 (1.13-1.33) | <0.001 |
| **Participation in Community Groups (No.)** | 1.27 (1.19-1.36) | <0.001 | 1.00 (0.95-1.05) | 0.945 |
| **Loneliness** | 1.02 (0.98-1.06) | 0.310 | 1.00 (0.97-1.04) | 0.757 |
| **Distance to Meetings in Village (km)** | 0.63 (0.45-0.89) | 0.008 | 0.56 (0.39-0.80) | 0.001 |
| **Constant** |  |  | 0.12 (0.08-0.18) | <0.001 |
| **Observations** |  | | 1,524 | |
| Abbreviations: RR, relative risk; ARR, adjusted relative risk; CI, confidence interval  The adjusted model includes each of the variables listed in the table row headers: age, sex, marital status, education, HIV status, obesity, depression symptom severity, household food insecurity, household water insecurity, household asset wealth, membership in community groups, participation in community groups, loneliness, and straight-line distance to the meeting corresponding to participant’s village of residence | | | | |

Given our justification above for not analyzing the data in this way, and also given that we believe this analysis does not lend additional insight, we have elected to exclude this analysis from the manuscript. However, at the reviewer’s or editor’s request, we would be happy to include it in the manuscript along with a substantive discussion, or perhaps include it in the manuscript as a Supporting Information Table.

**Summary:**

**Mostly points of detail.**

**Michael Dewey**

**References:**

**D G Altman and P Royston. The cost of dichotomising continuous variables. *British Medical Journal,* 332:1080, 2006.**

**P Royston, D G Altman, and W Sauerbrei. Dichotomizing continuous predictors in multiple regression: a bad idea. *Statistics in Medicine*, 25:127-141, 2006.**

**Reviewer #2:**

**This is an interesting research, that adopts an original approach for a better understanding of community engagement (an important emerging theme in research ethics – particularly but not only in global health research). It is supported by a comprehensive literature search. I have a few minor comments:**

RESPONSE: We thank the reviewer for the kind comments. We hope the revisions, described in depth below, address the stated concerns.

**1. I understand that for the work presented in this manuscript, there was no prospective data collection, and only secondary data from the social network study were used. The names of those who attended the community meetings were linked to the pseudonymized data from a community survey conducted in 2016-2018 with 1630 participants (including sensitive health information, GPS positions etc.). Participants who did not attend a community sensitization meeting but who may have been indirectly exposed to a meeting were also retro-identified through their social affiliation with someone who did attend a meeting. Given that researchers accessed retrospective and identifiable personal data, it would be important to mention if the initial consent for the community survey included use in (this) future research, or if attendants of the community meetings consented to link their signature to the stored personal data.**

RESPONSE: Participants provided written informed consent prior to participating in the community survey and community health fair. The sociocentric (whole-network) social network design of the study involves eliciting from each study participant the names of his or her significant social ties who live within the study site catchment area. Prior to conducting the survey, our team conducts an extensive census of the study site catchment area, enumerating all households, all individuals, and all potentially eligible study participants. Thus, by design, each social tie who is nominated is also a study participant who has provided informed consent to participate. During the informed consent process, potential study participants are educated about the study design and about the fact that their data will be linked to others through social affiliations. Finally, protected health information – including names, phone numbers, and GPS locations of household residences – are used for a number of purposes, including linking datasets and to enhance participant follow-up and retention.

**2. The authors often refers to "geodesic distance", thus it would be good to include a definition of this term in the context of this manuscript.**

RESPONSE: After first mentioning “geodesic distance” in the Data Analysis subsection of the Methods, we define this term.

“We identified the number of study participants who did not attend a community sensitization meeting themselves and who were a geodesic distance of 1 from meeting attendees [73]. Formally, the geodesic distance between two vertices in a network graph is the number of edges corresponding to the shortest path connecting the two vertices; informally, a geodesic distance of 1 is one handshake away.” (page 18)

**3. It is not fully clear if the community meetings took place before or after the social network protocol finalization and ethics approval, and to what extent the feedback of the community led to changes in the study design, as suggested in Figure 1. Even if not related to the primary outcome of this manuscript, it would be interesting to clarify this, and to provide any examples of community-prompted changes, for a better understanding on whether there was actual co-design of the research.**

RESPONSE: The study is a longitudinal, sociocentric social network study. Prior to commencing the study in 2014, we first obtained feedback from a community advisory board comprised of eight community leaders, including four women and the district community development officer. Their feedback was incorporated into the study design, and the protocol (which described our plans to conduct community sensitization meetings) was submitted for ethics approval. After ethics approval, we commenced the first community sensitization meetings in advance of the first community survey and the first community health fair. We then held sensitization meetings prior to subsequent waves of the community survey and community health fair. Given the longitudinal study design, concerns and recommendations expressed during these meetings are integrated into future study activities through IRB amendment applications or in the design of future community survey waves or community health fairs. For example, in 2017 we incorporated high-risk HPV testing and cervical cancer screening into the community health fair in response to requests from community members and community leaders. We elaborate in the Methods section.

“Prior to commencing the study, we obtained feedback from a CAB comprised of eight community leaders, including four women and the district community development officer. Their feedback was incorporated into the study design, and the study protocol was reviewed and approved by the Mbarara University of Science and Technology Research Ethics Committee and the Partners Human Research Committee. Consistent with Ugandan national guidelines, clearance for the study was also obtained from the Uganda National Council for Science and Technology. Upon receiving approval, we began conducting community sensitization meetings. Additional community sensitization meetings were held prior to subsequent waves of the community surveys and the community health fairs, thus providing opportunities for community input to be incorporated into subsequent research study activities. For example, during a community sensitization meeting held prior to the first community health fair, meeting attendees requested that our study team provide cervical cancer screening as part of the community health fair activities. In response, we incorporated into the subsequent community health fair a program of cervical cancer prevention education, high-risk human papillomavirus testing, and screening for premalignant lesions using visual inspection with acetic acid (with cryotherapy for screen-positive women meeting treatment criteria). For all study activities, participants provided written informed consent prior to participation; those who could not read and/or write were permitted to indicate consent with a thumbprint mark.” (page 12-13)

**4. The authors indicate interesting lines for future research, including triangulation of qualitative and quantitative data. Perhaps, they could also briefly reflect on whether/how such future research could be codesigned with the community.**

RESPONSE: We thank the reviewer for this recommendation. We have updated the first paragraph of the Strengths and Limitations subsection of the Discussion to describe how mixed methods research might be codesigned with the community.

“Future research in this area could be codesigned with community members. For example, focus groups could be used to elicit community members’ perceptions about why some individuals may or may not attend community sensitization meetings. This feedback could then be integrated into community surveys or the development of key informant interview guides.” (page 32)

**Reviewer #3:**

**This well-written paper addresses a critical topic in health research studies - community sensitization and how well strategies to educate and engage the community in upcoming research actually reach the target population. Their main findings are that older, married, women are the most likely to attend sensitization meetings, and that through their social network and household connections a larger proportion of the population can be reached. However, there are still under-represented groups that may need more specific targeting.**

**Overall, the paper is well-written, and the analyses are appropriate. Some minor comments below:**

RESPONSE: We thank the reviewer for the positive feedback on our manuscript. We attend to these comments below and in the text.

**1. Were there any differences in demographics between the group that attended community sensitization before the survey and those who attended before the health fairs? What percent attended both?**

RESPONSE: At the beginning of the Results section we clarify that 10% of the study participants (170/1,630) attended at least one meeting before both study activities.

“Of these participants, 264 (16%) attended a community sensitization meeting before the community survey, 464 (28%) attended a meeting before the community health fair, 558 (34%) attended a meeting before either study activity (survey and/or health fair), and 170 (10%) attended a meeting before both study activities (survey and health fair).” (page 18-19)

We also provide a brief demographic comparison of participants in the full sample to participants who attended a community sensitization meeting before the two different study activities. Additional demographic comparison of attendees and non-attendees of community sensitization meetings held in advance of the community survey and the community health fair are provided in the Supporting Information Files (S1 Table and S2 Table).

“Compared to the full sample of participants (mean age=39.4 years; standard deviation [SD]=16.7), participants who attended community sensitization meetings were older (community survey: mean age=45.1; SD=15.6; community health fair: mean age=44.7; SD=15.5). Additionally, a higher percentage of meeting attendees were women (overall sample, 56%; community survey meeting attendees, 65%; community health fair meeting attendees, 70%) and married (overall sample, 61%; community survey meeting attendees, 75%; community health fair meeting attendees, 70%).” (page 21)

**2. Can more information be provided about the participants' network position and network characteristics? Specifically, the centrality (most interesting would be in-degree and out-degree, betweenness, and closeness) should be straightforward to present in the sociocentric networks.**

RESPONSE: Information on participants’ network position and network characteristics (in-degree, out-degree, closeness, and betweenness) is now presented in Table 2, S1 Table, and S2 Table.

In the section of the Results on “Expanded Reach of Community Sensitization Meetings Beyond Attendees”, we note that non-attendees in attendees’ social networks were more socially integrated than non-attendees not in attendees’ social networks, across network measures. We also not that non-attendees in attendees’ households were less socially integrated than attendees, across network measures.

“Comparing in-degree, out-degree, closeness centrality, and betweenness centrality, non-attendees (both for the community survey and the community health fair meetings) in attendees’ social networks were more socially integrated compared with non-attendees not in attendees’ social networks.” (page 25)

“Comparing in-degree, out-degree, closeness centrality, and betweenness centrality (both for the community survey and the community health fair meetings), non-attendees in attendees’ households were less socially integrated than attendees.” (page 27)

Additionally, in a regression analysis that we include in this response memo only, we included the network measures in the regression models along with the other covariates. Across specifications, in-degree had a statistically significant association with attendance at community sensitization meetings held before the community survey and the community health fair. The other network measures had statistically significant bivariate associations with the outcomes but did not have statistically significant associations once all covariates were included in the regression models.

**Reviewer Table 3.** Unadjusted and multivariable Poisson regression models estimating correlates of attendance at community sensitization meetings.

*Note: Estimates correspond to the Poisson regression estimates in Table 3 of the manuscript, with centrality measures (in-degree, out-degree, closeness, betweenness) included in the regression models.*

|  | **Attendance at Community Sensitization Meeting Held in Advance of Community Survey** | | | | **Attendance at Community Sensitization Meeting Held in Advance of Community Health Fair** | | | |
| --- | --- | --- | --- | --- | --- | --- | --- | --- |
|  | **RR**  **(95% CI)** | ***p*-value** | **ARR**  **(95% CI)** | ***p*-value** | **RR**  **(95% CI)** | ***p*-value** | **ARR**  **(95% CI)** | ***p*-value** |
| **Age** | 1.02  (1.01-1.02) | <0.001 | 1.01  (1.01-1.02) | <0.001 | 1.02  (1.01-1.02) | <0.001 | 1.01  (1.01-1.02) | <0.001 |
| **Female** | 1.45  (1.10-1.91) | 0.009 | 1.40  (1.03-1.90) | 0.033 | 1.85  (1.41-2.41) | <0.001 | 1.77  (1.39-2.24) | <0.001 |
| **Married** | 1.79  (1.31-2.46) | <0.001 | 1.65  (1.22-2.25) | 0.001 | 1.48  (1.20-1.83) | <0.001 | 1.32  (1.05-1.66) | 0.016 |
| **Completed Primary School** | 0.68  (0.53-0.88) | 0.003 | 0.97  (0.80-1.17) | 0.759 | 0.71  (0.58-0.86) | <0.001 | 1.06  (0.87-1.30) | 0.548 |
| **HIV Positive** | 1.13  (0.66-1.94) | 0.662 | 1.06  (0.53-2.09) | 0.874 | 1.25  (0.93-1.69) | 0.144 | 1.06  (0.83-1.35) | 0.667 |
| **Obese** | 1.20  (0.92-1.56) | 0.175 | 0.92  (0.70-1.19) | 0.512 | 1.35  (1.11-1.65) | 0.003 | 0.98  (0.85-1.14) | 0.821 |
| **Depression Symptom Severity** | 1.37  (1.14-1.65) | 0.001 | 1.30  (1.04-1.64) | 0.023 | 1.25  (1.10-1.43) | 0.001 | 1.07  (0.92-1.24) | 0.384 |
| **Household Food Insecurity:**  Food Secure (*ref*) |  |  |  |  |  |  |  |  |
| Mild Food Insecurity | 1.16  (0.79-1.70) | 0.451 | 0.90  (0.55-1.47) | 0.665 | 0.98  (0.70-1.35) | 0.881 | 0.89  (0.67-1.18) | 0.399 |
| Moderate Food Insecurity | 1.28  (0.73-2.25) | 0.381 | 0.99  (0.55-1.81) | 0.983 | 1.30  (0.99-1.70) | 0.060 | 1.09  (0.87-1.38) | 0.456 |
| Severe Food Insecurity | 1.22  (0.76-1.95) | 0.402 | 0.94  (0.57-1.53) | 0.790 | 1.28  (1.02-1.59) | 0.030 | 1.11  (0.91-1.36) | 0.316 |
| **Household Water Insecurity:**  Water Secure (*ref*) |  |  |  |  |  |  |  |  |
| Mild Water Insecurity | 1.30  (0.99-1.70) | 0.056 | 1.11  (0.95-1.30) | 0.194 | 1.06  (0.89-1.26) | 0.485 | 0.96  (0.85-1.09) | 0.509 |
| Moderate Water Insecurity | 1.07  (0.70-1.64) | 0.754 | 0.97  (0.67-1.40) | 0.852 | 1.11  (0.95-1.30) | 0.199 | 1.01  (0.88-1.15) | 0.906 |
| Severe Water Insecurity | 0.91  (0.63-1.31) | 0.594 | 0.83  (0.66-1.06) | 0.133 | 1.01  (0.81-1.26) | 0.923 | 0.93  (0.79-1.10) | 0.415 |
| **Household Asset Wealth Quintile Category**  1st (Poorest, *ref*) |  |  |  |  |  |  |  |  |
| 2nd | 1.17  (0.79-1.71) | 0.434 | 1.06  (0.68-1.66) | 0.801 | 1.01  (0.86-1.19) | 0.906 | 0.93  (0.80-1.08) | 0.354 |
| 3rd | 1.09  (0.91-1.30) | 0.357 | 0.94  (0.74-1.19) | 0.607 | 1.04  (0.96-1.12) | 0.320 | 0.92  (0.77-1.10) | 0.352 |
| 4th | 1.00  (0.85-1.16) | 0.957 | 0.77  (0.55-1.08) | 0.130 | 0.92  (0.76-1.11) | 0.393 | 0.75  (0.60-0.95) | 0.018 |
| 5th (Least poor) | 0.62  (0.37-1.05) | 0.079 | 0.52  (0.30-0.92) | 0.026 | 0.58  (0.41-0.81) | 0.001 | 0.52  (0.37-0.74) | <0.001 |
| **Membership in Community Groups (No.)** | 1.34  (1.26-1.43) | <0.001 | 1.19  (1.05-1.35) | 0.005 | 1.33  (1.23-1.43) | <0.001 | 1.17  (1.06-1.30) | 0.003 |
| **Participation in Community Groups (No.)** | 1.34  (1.21-1.47) | <0.001 | 1.00  (0.83-1.21) | 1.000 | 1.31  (1.22-1.40) | <0.001 | 0.98  (0.90-1.07) | 0.699 |
| **Loneliness** | 1.01  (0.93-1.10) | 0.796 | 1.00  (0.92-1.09) | 0.937 | 1.01  (0.98-1.05) | 0.377 | 1.00  (0.97-1.03) | 0.853 |
| **Distance to Meetings in Village (km)** | 0.51  (0.28-0.93) | 0.028 | 0.60  (0.33-1.06) | 0.078 | 0.56  (0.39-0.80) | 0.002 | 0.66  (0.39-1.12) | 0.124 |
| **In-Degree** | 1.07  (1.05-1.08) | <0.001 | 1.04  (1.02-1.06) | <0.001 | 1.06  (1.05-1.07) | <0.001 | 1.02  (1.02-1.03) | <0.001 |
| **Out-Degree** | 1.09  (1.06-1.11) | <0.001 | 1.02  (0.99-1.05) | 0.163 | 1.09  (1.06-1.11) | <0.001 | 1.02  (0.99-1.06) | 0.199 |
| **Closeness Centrality** | 225513  (2.11-2.41e+10) | 0.037 | 1.65  (0.02-135) | 0.823 | 2164122  (11625-4.03e+8) | <0.001 | 93.9  (1.83-4831) | 0.024 |
| **Betweenness Centrality** | 1.00001  (1.00001-1.00002) | <0.001 | 1.000001  (1.00-1.00001) | 0.489 | 1.00001  (1.00001-1.00002) | <0.001 | 1.00  (1.00-1.00001) | 0.904 |
| **Constant** |  |  | 0.03  (0.02-0.06) | <0.001 |  |  | 0.03  (0.01-0.05) | <0.001 |
| **Observations** |  | | 1,294 | |  | | 1,519 | |
| Abbreviations: RR, relative risk; ARR, adjusted relative risk; CI, confidence interval  The adjusted models include each of the variables listed in the table row headers: age, sex, marital status, education, HIV status, obesity, depression symptom severity, household food insecurity, household water insecurity, household asset wealth, membership in community groups, participation in community groups, loneliness, and straight-line distance to the meeting corresponding to participant’s village of residence | | | | | | | | |

**3. Similarly, data on the composition of the participants' networks would also be informative. Specifically, household size and demographics of 1st degree contacts.**

RESPONSE: In the Supporting Information Files (S1 Table and S2 Table), we include the sociodemographic characteristics of meeting attendees and non-attendees. Of the non-attendees, we also include breakdowns of the non-attendees who are in attendees’ social networks and households.
