# Supplementary material for: Correlates of attendance at community engagement meetings held in advance of bio-behavioral research studies: A longitudinal, sociocentric social network study in rural Uganda
Source: PLoS Med. 2021 Jul 16;18(7):e1003705. doi: 10.1371/journal.pmed.1003705 (PMC8323877; doi:10.1371/journal.pmed.1003705)
Supplement: Attachment — Submitted filename: commsens_plosmed_r2_20210617_response.docx [file pmed.1003705.s007.docx]

**Requests from Editors:**

**1. Data availability statement: Thank you for your willingness to make your data available. At this time, please provide the link to access the anonymized data and Stata code through an online repository.**

RESPONSE: We have uploaded the anonymized data and code to GitHub. On the cover page, we have added the following statement:

“Data Availability: All files are available from the following GitHub repository: [https://github.com/esatinsky/commsen](https://github.com/esatinsky/communitysensitization_paper)s”

**2. Abstract: Line 22: There appears to be a typo in reporting the p value for the adjusted relative risk for women attending meetings ahead of the survey: [ARR]survey=1.38, 95% confidence interval [CI], 0.99-1.92, p=0.55. In the results, the p value is noted to be p=0.055. This is described in the results as the women being no more likely to attend meetings held before the survey- noting that the difference does not reach statistical significance, if possible, please describe the result in the abstract such that the interpretation matches the results.**

RESPONSE: To remain within *PLoS Medicine’s* Abstract word limit, we no longer report the statistics for the association between sex and attendance at a community sensitization meeting held before the survey. We have also confirmed that the statistics reported in the Abstract match those reported in the Results.

**3. Figure 2: Please confirm that the appropriate usage rights apply to the use of this map. Please see our guidelines for map images: https://journals.plos.org/plosmedicine/s/figures#loc-maps**

RESPONSE: The background image for the map in Figure 2 is from OpenStreetMap in ArcGIS. We are uploading an updated Figure file with the following attribution statement:

“*Contains information from OpenStreetMap and OpenStreetMap Foundation, which is made available under the Open Database License”

**4. Table 2: Please define all abbreviations in the legend (IQR).**

RESPONSE: We now define IQR in the legend at the bottom of Table 2.

**5. Acknowledgements: Please remove the trademark designation (TM) for the software.**

RESPONSE: We have removed the trademark designation from the Acknowledgements.

**6. Supporting information files: Please include the STROBE S1 Checklist and S1 Text, and Tables as separate files. For S1 and S2 Tables, please spell out all abbreviations in the legend (IQR).**

RESPONSE: We have created separate documents for each supporting information file. S1 and S2 Tables now include abbreviations in the legends.

**Comments from Reviewers:

Reviewer #1: The authors have addressed all my points.

Michael Dewey**

RESPONSE: We thank the reviewer for taking the time to review our manuscript.
