# Supplementary material for: Correlates of attendance at community engagement meetings held in advance of bio-behavioral research studies: A longitudinal, sociocentric social network study in rural Uganda
Source: PLoS Med. 2021 Jul 16;18(7):e1003705. doi: 10.1371/journal.pmed.1003705 (PMC8323877; doi:10.1371/journal.pmed.1003705)
Supplement: Peer review history

# Editor report: Decision Letter 0

**DOI:** 10.1371/journal.pmed.1003705.r001

**Authors:** Caitlin Moyer

26 Jan 2021

Dear Dr Satinsky, 

Thank you for submitting your manuscript entitled "Who attends community sensitization meetings to provide input on human subjects research studies? Evidence from a sociocentric social network cohort in rural Uganda" for consideration by PLOS Medicine.

Your manuscript has now been evaluated by the PLOS Medicine editorial staff as well as by an academic editor with relevant expertise and I am writing to let you know that we would like to send your submission out for external peer review.

However, before we can send your manuscript to reviewers, we need you to complete your submission by providing the metadata that is required for full assessment. To this end, please login to Editorial Manager where you will find the paper in the 'Submissions Needing Revisions' folder on your homepage. Please click 'Revise Submission' from the Action Links and complete all additional questions in the submission questionnaire.

Please re-submit your manuscript within two working days, i.e. by .

Login to Editorial Manager here: https://www.editorialmanager.com/pmedicine

Once your full submission is complete, your paper will undergo a series of checks in preparation for peer review. Once your manuscript has passed all checks it will be sent out for review. 

Feel free to email us at plosmedicine@plos.org if you have any queries relating to your submission.

Kind regards,

Caitlin Moyer, Ph.D.

Associate Editor

PLOS Medicine

# Peer review: Decision Letter 1

**DOI:** 10.1371/journal.pmed.1003705.r002

**Authors:** Caitlin Moyer

9 Apr 2021

Dear Dr. Satinsky,

Thank you very much for submitting your manuscript "Who attends community sensitization meetings to provide input on human subjects research studies? Evidence from a sociocentric social network cohort in rural Uganda" (PMEDICINE-D-21-00131R1) for consideration at PLOS Medicine. 

Your paper was evaluated by a senior editor and discussed among all the editors here. It was also discussed with an academic editor with relevant expertise, and sent to three independent reviewers, including a statistical reviewer. The reviews are appended at the bottom of this email and any accompanying reviewer attachments can be seen via the link below:

[LINK]

In light of these reviews, I am afraid that we will not be able to accept the manuscript for publication in the journal in its current form, but we would like to consider a revised version that addresses the reviewers' and editors' comments. Obviously we cannot make any decision about publication until we have seen the revised manuscript and your response, and we plan to seek re-review by one or more of the reviewers. 

In revising the manuscript for further consideration, your revisions should address the specific points made by each reviewer and the editors. Please also check the guidelines for revised papers at http://journals.plos.org/plosmedicine/s/revising-your-manuscript for any that apply to your paper. In your rebuttal letter you should indicate your response to the reviewers' and editors' comments, the changes you have made in the manuscript, and include either an excerpt of the revised text or the location (eg: page and line number) where each change can be found. Please submit a clean version of the paper as the main article file; a version with changes marked should be uploaded as a marked up manuscript.

In addition, we request that you upload any figures associated with your paper as individual TIF or EPS files with 300dpi resolution at resubmission; please read our figure guidelines for more information on our requirements: http://journals.plos.org/plosmedicine/s/figures. While revising your submission, please upload your figure files to the PACE digital diagnostic tool, https://pacev2.apexcovantage.com/. PACE helps ensure that figures meet PLOS requirements. To use PACE, you must first register as a user. Then, login and navigate to the UPLOAD tab, where you will find detailed instructions on how to use the tool. If you encounter any issues or have any questions when using PACE, please email us at PLOSMedicine@plos.org.

We expect to receive your revised manuscript by Apr 30 2021 11:59PM. Please email us (plosmedicine@plos.org) if you have any questions or concerns.

***Please note while forming your response, if your article is accepted, you may have the opportunity to make the peer review history publicly available. The record will include editor decision letters (with reviews) and your responses to reviewer comments. If eligible, we will contact you to opt in or out.***

We ask every co-author listed on the manuscript to fill in a contributing author statement, making sure to declare all competing interests. If any of the co-authors have not filled in the statement, we will remind them to do so when the paper is revised. If all statements are not completed in a timely fashion this could hold up the re-review process. If new competing interests are declared later in the revision process, this may also hold up the submission. Should there be a problem getting one of your co-authors to fill in a statement we will be in contact. YOU MUST NOT ADD OR REMOVE AUTHORS UNLESS YOU HAVE ALERTED THE EDITOR HANDLING THE MANUSCRIPT TO THE CHANGE AND THEY SPECIFICALLY HAVE AGREED TO IT. You can see our competing interests policy here: http://journals.plos.org/plosmedicine/s/competing-interests.

Please use the following link to submit the revised manuscript: 

https://www.editorialmanager.com/pmedicine/

Your article can be found in the "Submissions Needing Revision" folder. 

To enhance the reproducibility of your results, we recommend that you deposit your laboratory protocols in protocols.io, where a protocol can be assigned its own identifier (DOI) such that it can be cited independently in the future. Additionally, PLOS ONE offers an option to publish peer-reviewed clinical study protocols. Read more information on sharing protocols at https://plos.org/protocols?utm_medium=editorial-email&utm_source=authorletters&utm_campaign=protocols

Please ensure that the paper adheres to the PLOS Data Availability Policy (see http://journals.plos.org/plosmedicine/s/data-availability), which requires that all data underlying the study's findings be provided in a repository or as Supporting Information. For data residing with a third party, authors are required to provide instructions with contact information for obtaining the data. PLOS journals do not allow statements supported by "data not shown" or "unpublished results." For such statements, authors must provide supporting data or cite public sources that include it. 

We look forward to receiving your revised manuscript. 

Sincerely,

Caitlin Moyer, PhD

Associate Editor

PLOS Medicine

plosmedicine.org

-----------------------------------------------------------

From the Academic Editor: 

The one thing is that is needed, from my perspective, is more information and context of social dynamics between the village health teams and other "gatekeepers" and the different segments of the community.

The implication is that this mechanism is one that allows people to participate and have voice, and is likely to be the one in which other health program or research access the community voice as well.

Would like to see some more discussion about implications and theory linking this practice to governance of studies and health programs. There are clearly voices in communities that have more or less access - does this need to remedied and if so how?

What implications for research? public health programs? What about studies and programs that rely on these networks to inform design? Are there community equity issues?

Requests from the editors:

1. Title: Please revise your title according to PLOS Medicine's style. Your title must be nondeclarative and not a question. It should begin with main concept if possible. "Effect of" should be used only if causality can be inferred, i.e., for an RCT. Please place the study design ("A randomized controlled trial," "A retrospective study," "A modelling study," etc.) in the subtitle (ie, after a colon).

2. Abstract: Methods and Findings: Please include the study design, population and setting, number of participants, years during which the study took place, length of follow up, and main outcome measures. Please quantify the main results (with 95% CIs and p values). As the last sentence of the Abstract Methods and Findings section, please describe the main limitation(s) of the study's methodology.

3. Abstract: Conclusions: Please include a sentence interpreting the study’s key findings; the phrase "In this study, we observed ..." may be useful.

4. Author Summary: At this stage, we ask that you include a short, non-technical Author Summary of your research to make findings accessible to a wide audience that includes both scientists and non-scientists. The Author Summary should immediately follow the Abstract in your revised manuscript. This text is subject to editorial change and should be distinct from the scientific abstract. Please see our author guidelines for more information: https://journals.plos.org/plosmedicine/s/revising-your-manuscript#loc-author-summary

5. Methods: Please ensure that the study is reported according to the STROBE guideline, and include the completed STROBE checklist as Supporting Information. Please add the following statement, or similar, to the Methods: "This study is reported as per the Strengthening the Reporting of Observational Studies in Epidemiology (STROBE) guideline (S1 Checklist)."

The STROBE guideline can be found here: http://www.equator-network.org/reporting-guidelines/strobe/

When completing the checklist, please use section and paragraph numbers, rather than page numbers.

6. Methods: Did your study have a prospective protocol or analysis plan? Please state this (either way) early in the Methods section.

a) If a prospective analysis plan (from your funding proposal, IRB or other ethics committee submission, study protocol, or other planning document written before analyzing the data) was used in designing the study, please include the relevant prospectively written document with your revised manuscript as a Supporting Information file to be published alongside your study, and cite it in the Methods section. A legend for this file should be included at the end of your manuscript. 

b) If no such document exists, please make sure that the Methods section transparently describes when analyses were planned, and when/why any data-driven changes to analyses took place. 

c) In either case, changes in the analysis-- including those made in response to peer review comments-- should be identified as such in the Methods section of the paper, with rationale.

7. Methods: Please clarify the years during which the study took place.

8. Methods: Is it a potential limitation of the study that the linked survey data were from 2016-2018 while the attendance information was from 2018-2020 (potentially out of date social network, health, demographic data)?

9. Table 3: Please note the variables used in adjustment in the legend.

10. References: Please use the "Vancouver" style for reference formatting (including NLM journal name abbreviations), and see our website for other reference guidelines https://journals.plos.org/plosmedicine/s/submission-guidelines#loc-references

Comments from the reviewers:

Reviewer #1: See attachment

Michael Dewey

Reviewer #2: This is an interesting research, that adopts an original approach for a better understanding of community engagement (an important emerging theme in research ethics - particularly but not only in global health research). It is supported by a comprehensive literature search. I have a few minor comments: 

1. I understand that for the work presented in this manuscript, there was no prospective data collection, and only secondary data from the social network study were used. The names of those who attended the community meetings were linked to the pseudonymized data from a community survey conducted in 2016-2018 with 1630 participants (including sensitive health information, GPS positions etc.). Participants who did not attend a community sensitization meeting but who may have been indirectly exposed to a meeting were also retro-identified through their social affiliation with someone who did attend a meeting. Given that researchers accessed retrospective and identifiable personal data, it would be important to mention if the initial consent for the community survey included use in (this) future research, or if attendants of the community meetings consented to link their signature to the stored personal data

2. The authors often refers to "geodesic distance", thus it would be good to include a definition of this term in the context of this manuscript

3. It is not fully clear if the community meetings took place before or after the social network protocol finalization and ethics approval, and to what extent the feedback of the community led to changes in the study design, as suggested in Figure 1. Even if not related to the primary outcome of this manuscript, it would be interesting to clarify this, and to provide any examples of community-prompted changes, for a better understanding on whether there was actual co-design of the research. 

4. The authors indicate interesting lines for future research, including triangulation of qualitative and quantitative data. Perhaps, they could also briefly reflect on whether/how such future research could be codesigned with the community. 

Reviewer #3: This well-written paper addresses a critical topic in health research studies - community sensitization and how well strategies to educate and engage the community in upcoming research actually reach the target population. Their main findings are that older, married, women are the most likely to attend sensitization meetings, and that through their social network and household connections a larger proportion of the population can be reached. However there are still under-represented groups that may need more specific targeting.

Overall the paper is well-written and the analyses are appropriate. Some minor comments below:

1. Were there any differences in demographics between the group that attended community sensitization before the survey and those who attended before the health fairs? What percent attended both?

2. Can more information be provided about the participants' network position and network characteristics? Specifically, the centrality (most interesting would be in-degree and out-degree, betweenness, and closeness) should be straightforward to present in the sociocentric networks.

3. Similarly, data on the composition of the participants' networks would also be informative. Specifically, household size and demographics of 1st degree contacts.

Any attachments provided with reviews can be seen via the following link:

[LINK]

Attachment Submitted filename: kakuhikire.pdf Click here for additional data file.

# Author comment: Author response to Decision Letter 1

**DOI:** 10.1371/journal.pmed.1003705.r003

11 May 2021

Attachment Submitted filename: commsensitiz_response-to-reviewers_plosmed_20210508.docx Click here for additional data file.

# Peer review: Decision Letter 2

**DOI:** 10.1371/journal.pmed.1003705.r004

**Authors:** Caitlin Moyer

11 Jun 2021

Dear Dr. Satinsky,

Thank you very much for re-submitting your manuscript "Correlates of attendance at community engagement meetings held in advance of bio-behavioral research studies: A longitudinal, sociocentric social network study in rural Uganda" (PMEDICINE-D-21-00131R2) for review by PLOS Medicine.

I have discussed the paper with my colleagues and the academic editor and it was also seen again by one of the reviewers. I am pleased to say that provided the remaining editorial and production issues are dealt with we are planning to accept the paper for publication in the journal.

The remaining issues that need to be addressed are listed at the end of this email. Any accompanying reviewer attachments can be seen via the link below. Please take these into account before resubmitting your manuscript:

[LINK]

***Please note while forming your response, if your article is accepted, you may have the opportunity to make the peer review history publicly available. The record will include editor decision letters (with reviews) and your responses to reviewer comments. If eligible, we will contact you to opt in or out.***

In revising the manuscript for further consideration here, please ensure you address the specific points made by each reviewer and the editors. In your rebuttal letter you should indicate your response to the reviewers' and editors' comments and the changes you have made in the manuscript. Please submit a clean version of the paper as the main article file. A version with changes marked must also be uploaded as a marked up manuscript file.

Please also check the guidelines for revised papers at http://journals.plos.org/plosmedicine/s/revising-your-manuscript for any that apply to your paper. If you haven't already, we ask that you provide a short, non-technical Author Summary of your research to make findings accessible to a wide audience that includes both scientists and non-scientists. The Author Summary should immediately follow the Abstract in your revised manuscript. This text is subject to editorial change and should be distinct from the scientific abstract.

We expect to receive your revised manuscript within 1 week. Please email us (plosmedicine@plos.org) if you have any questions or concerns.

We ask every co-author listed on the manuscript to fill in a contributing author statement. If any of the co-authors have not filled in the statement, we will remind them to do so when the paper is revised. If all statements are not completed in a timely fashion this could hold up the re-review process. Should there be a problem getting one of your co-authors to fill in a statement we will be in contact. YOU MUST NOT ADD OR REMOVE AUTHORS UNLESS YOU HAVE ALERTED THE EDITOR HANDLING THE MANUSCRIPT TO THE CHANGE AND THEY SPECIFICALLY HAVE AGREED TO IT.

Please ensure that the paper adheres to the PLOS Data Availability Policy (see http://journals.plos.org/plosmedicine/s/data-availability), which requires that all data underlying the study's findings be provided in a repository or as Supporting Information. For data residing with a third party, authors are required to provide instructions with contact information for obtaining the data. PLOS journals do not allow statements supported by "data not shown" or "unpublished results." For such statements, authors must provide supporting data or cite public sources that include it.

To enhance the reproducibility of your results, we recommend that you deposit your laboratory protocols in protocols.io, where a protocol can be assigned its own identifier (DOI) such that it can be cited independently in the future. Additionally, PLOS ONE offers an option to publish peer-reviewed clinical study protocols. Read more information on sharing protocols at https://plos.org/protocols?utm_medium=editorial-email&utm_source=authorletters&utm_campaign=protocols

Please review your reference list to ensure that it is complete and correct. If you have cited papers that have been retracted, please include the rationale for doing so in the manuscript text, or remove these references and replace them with relevant current references. Any changes to the reference list should be mentioned in the rebuttal letter that accompanies your revised manuscript.

Please note, when your manuscript is accepted, an uncorrected proof of your manuscript will be published online ahead of the final version, unless you've already opted out via the online submission form. If, for any reason, you do not want an earlier version of your manuscript published online or are unsure if you have already indicated as such, please let the journal staff know immediately at plosmedicine@plos.org.

If you have any questions in the meantime, please contact me or the journal staff on plosmedicine@plos.org.  

We look forward to receiving the revised manuscript by Jun 18 2021 11:59PM.   

Sincerely,

Caitlin Moyer, Ph.D.

Associate Editor 

PLOS Medicine

plosmedicine.org

------------------------------------------------------------

Requests from Editors:

1. Data availability statement: Thank you for your willingness to make your data available. At this time, please provide the link to access the anonymized data and Stata code through an online repository.

2. Abstract: Line 22: There appears to be a typo in reporting the p value for the adjusted relative risk for women attending meetings ahead of the survey: [ARR]survey=1.38, 95% confidence interval [CI], 0.99-1.92, p=0.55. In the results, the p value is noted to be p=0.055. This is described in the results as the women being no more likely to attend meetings held before the survey- noting that the difference does not reach statistical significance, if possible please describe the result in the abstract such that the interpretation matches the results.

3. Figure 2: Please confirm that the appropriate usage rights apply to the use of this map. Please see our guidelines for map images: https://journals.plos.org/plosmedicine/s/figures#loc-maps

4. Table 2: Please define all abbreviations in the legend (IQR).

5. Acknowledgements: Please remove the trademark designation (TM) for the software.

6. Supporting information files: Please include the STROBE S1 Checklist and S1 Text, and Tables as separate files. For S1 and S2 Tables, please spell out all abbreviations in the legend (IQR).

Comments from Reviewers:

Reviewer #1: The authors have addressed all my points.

Michael Dewey

Any attachments provided with reviews can be seen via the following link:

[LINK]

# Author comment: Author response to Decision Letter 2

**DOI:** 10.1371/journal.pmed.1003705.r005

17 Jun 2021

Attachment Submitted filename: commsens_plosmed_r2_20210617_response.docx Click here for additional data file.

# Editor report: Decision Letter 3

**DOI:** 10.1371/journal.pmed.1003705.r006

**Authors:** Caitlin Moyer

21 Jun 2021

Dear Dr Satinsky, 

On behalf of my colleagues and the Academic Editor, Elvin Geng, I am pleased to inform you that we have agreed to publish your manuscript "Correlates of attendance at community engagement meetings held in advance of bio-behavioral research studies: A longitudinal, sociocentric social network study in rural Uganda" (PMEDICINE-D-21-00131R3) in PLOS Medicine.

Before your manuscript can be formally accepted you will need to complete some formatting changes, which you will receive in a follow up email. Please be aware that it may take several days for you to receive this email; during this time no action is required by you. Once you have received these formatting requests, please note that your manuscript will not be scheduled for publication until you have made the required changes.

In the meantime, please log into Editorial Manager at http://www.editorialmanager.com/pmedicine/, click the "Update My Information" link at the top of the page, and update your user information to ensure an efficient production process. 

PRESS

We frequently collaborate with press offices. If your institution or institutions have a press office, please notify them about your upcoming paper at this point, to enable them to help maximise its impact. If the press office is planning to promote your findings, we would be grateful if they could coordinate with medicinepress@plos.org. If you have not yet opted out of the early version process, we ask that you notify us immediately of any press plans so that we may do so on your behalf.

We also ask that you take this opportunity to read our Embargo Policy regarding the discussion, promotion and media coverage of work that is yet to be published by PLOS. As your manuscript is not yet published, it is bound by the conditions of our Embargo Policy. Please be aware that this policy is in place both to ensure that any press coverage of your article is fully substantiated and to provide a direct link between such coverage and the published work. For full details of our Embargo Policy, please visit http://www.plos.org/about/media-inquiries/embargo-policy/.

To enhance the reproducibility of your results, we recommend that you deposit your laboratory protocols in protocols.io, where a protocol can be assigned its own identifier (DOI) such that it can be cited independently in the future. Additionally, PLOS ONE offers an option to publish peer-reviewed clinical study protocols. Read more information on sharing protocols at https://plos.org/protocols?utm_medium=editorial-email&utm_source=authorletters&utm_campaign=protocols

Thank you again for submitting to PLOS Medicine. We look forward to publishing your paper. 

Sincerely, 

Caitlin Moyer, Ph.D. 

Associate Editor 

PLOS Medicine
